# Development of a colorimetric assay for the detection of SARS-CoV-2 3CLpro activity

**DOI:** 10.1042/BCJ20220105

**Published:** 2022-04-21

**Authors:** Gavin D. Garland, Robert F. Harvey, Thomas E. Mulroney, Mie Monti, Stewart Fuller, Richard Haigh, Pehuén Pereyra Gerber, Michael R. Barer, Nicholas J. Matheson, Anne E. Willis

**Affiliations:** 1 MRC Toxicology Unit, Gleeson Building, Tennis Court Rd, Cambridge, U.K.; 2 Department of Medicine, University of Cambridge, Cambridge, U.K.; 3 Department of Respiratory Sciences, Maurice Shock Medical Sciences Building, University Road, Leicester, U.K.; 4 Cambridge Institute of Therapeutic Immunology and Infectious Disease (CITIID), University of Cambridge, Cambridge, U.K.; 5 NHS Blood and Transplant, Cambridge, U.K.

**Keywords:** assay development, Coronavirus, COVID 19

## Abstract

Diagnostic testing continues to be an integral component of the strategy to contain the Severe Acute Respiratory Syndrome Coronavirus 2 (SARS-CoV-2) global pandemic, the causative agent of Coronavirus Disease 2019 (COVID-19). The SARS-CoV-2 genome encodes the 3C-like protease (3CLpro) which is essential for coronavirus replication. This study adapts an *in vitro* colorimetric gold nanoparticle (AuNP) based protease assay to specifically detect the activity of SARS-CoV-2 3CLpro as a purified recombinant protein and as a cellular protein exogenously expressed in HEK293T human cells. We also demonstrate that the specific sensitivity of the assay for SARS-CoV-2 3CLpro can be improved by use of an optimised peptide substrate and through hybrid dimerisation with inactive 3CLpro mutant monomers. These findings highlight the potential for further development of the AuNP protease assay to detect SARS-CoV-2 3CLpro activity as a novel, accessible and cost-effective diagnostic test for SARS-CoV-2 infection at the point-of-care. Importantly, this versatile assay could also be easily adapted to detect specific protease activity associated with other viruses or diseases conditions.

## Introduction

A novel species of coronavirus, Severe Acute Respiratory Syndrome Coronavirus 2 (SARS-CoV-2), is known to be the causative agent of Coronavirus Disease 2019 (COVID-19). Since its reported emergence in Wuhan (China), the virus has spread worldwide resulting in a global pandemic which, at the time of writing, has resulted in 410,565,868 confirmed cases of COVID-19 including 5,810,880 deaths according to the World Health Organisation [1]. However, where data are available, excess mortality over this period indicates that the true cost of this outbreak may be considerably higher due to failure to diagnose this disease in some cases, and through deaths from other causes that can be attributed to the general pandemic crisis conditions [2].

The immense cost of COVID-19 has been experienced globally, and while the development of a growing number of vaccines against SARS-CoV-2 offers hope, the emergence of SARS-CoV-2 variants may limit the efficacy of vaccination programmes [3]. In addition, while the introduction of some treatments such as Paxlovid, Molnupiravir and Sotrovimab have improved the mortality rate of this disease where available [4,5], other treatments such as Remdesivir have not shown consistent significant clinical benefit [6], and other monoclonal antibody-based treatments have demonstrated reduced efficacy with SARS-CoV-2 variants such as Omicron [7]. Therefore, COVID-19 remains a serious health problem around the world and there is an urgent need to better control the spread of the causative agent SARS-CoV-2 and restrict outbreaks of future variants [8].

It has become clear that an effective and coordinated testing strategy plays a critical role in containing SARS-CoV-2 and limiting the impact of COVID-19 [9]. Currently, the gold-standard diagnostic test for SARS-CoV-2 remains the Reverse-Transcription Polymerase Chain Reaction (RT-PCR) assay which detects the presence of viral RNA in patient specimens, which are often combined nasopharyngeal/oropharyngeal swab samples. While the accuracy and sensitivity of these assays are high, the samples need to be processed and analysed by highly trained staff in specialised laboratories. This can result in significant delays between sampling and test results, limits the capacity for testing and increases the financial burden of supporting an effective testing programme [9,10]. The development of other diagnostic assays which can be used in point-of-care settings have helped to overcome some of these problems, but these alternative tests also have other drawbacks. For example, lateral flow antigen immunochromatographic assays (which are the leading candidates for mass community testing) work by qualitatively detecting specific SARS-CoV-2 antigens from a patient specimen which is captured by a SARS-CoV-2 monoclonal antibody on the test device and which can be interpreted by eye or using a dedicated reader to give results within 15 min [11]. However, the reduced accuracy of such tests appears to be a major limitation with studies reporting large numbers of false negative results which would hinder efforts to control community transmission rates. For example, the Cochrane COVID-19 Diagnostic Test Accuracy Group reports that commercially available rapid antigen tests on average only correctly identified SARS-CoV-2 infection in 72% of symptomatic subjects and in 58% of subjects displaying no symptoms [12].

Therefore, there remains an urgent, unmet need to develop alternative sensitive diagnostic assays for rapid and accurate detection of SARS-CoV-2 viral infection, preferably at an early stage of infection before patients become transmissible or ill. Ideally, these tests would be conducted at point of care and would not require specialised laboratories or highly trained staff to process and analyse the samples; would be cost effective and easily integrated around the world in countries with well-funded healthcare systems to support the ongoing testing programmes as well as in countries without well-funded healthcare systems where diagnosis and control of COVID-19 is currently problematic [9].

In common with other species of coronavirus, the SARS-CoV-2 genome contains the Non-structural protein 5 (Nsp5) gene which encodes the 3C-like protease (3CLpro; also known as Main Protease or Mpro) which in combination with Papain-like protease (PLpro) is required to cleave the two large polyproteins encoded by the replicase gene into its 16 non-structural proteins as an early event in the infection cycle [13]. The 3CLpro enzymes show a highly conserved structure among various coronavirus species and are essential for viral replication [14]. These proteases also target well-defined substrate recognition sequences and no endogenous human protease with analogous cleavage specificity is known [15–17]. These attributes have made 3CLpro an attractive target for the development of anti-viral treatments [18]. However, these are also qualities that can potentially be exploited for the design of novel diagnostic assays to detect SARS-CoV-2 infection through 3CLpro activity.

In 2014, Chen et al. described the development of a novel *in vitro* colorimetric assay to specifically detect the protease activity of Trypsin and Matrix Metalloproteinase-2 (MMP-2) enzymes. The assay utilises a gold nanoparticle (AuNP) solution which is stabilised by a short peptide which can be cleaved by a specific protease resulting in aggregation of the AuNPs and a marked colour change from red to blue [19]. In this study, this colorimetric protease assay is adapted to specifically detect the SARS-CoV-2 3CLpro enzyme and its potential use as a diagnostic assay for the detection of SARS-CoV-2 infection is investigated.

## Results

### SARS-CoV-2 3CLpro displays protease activity in the 3CLpro AuNP protease assay

**Figure 1.**
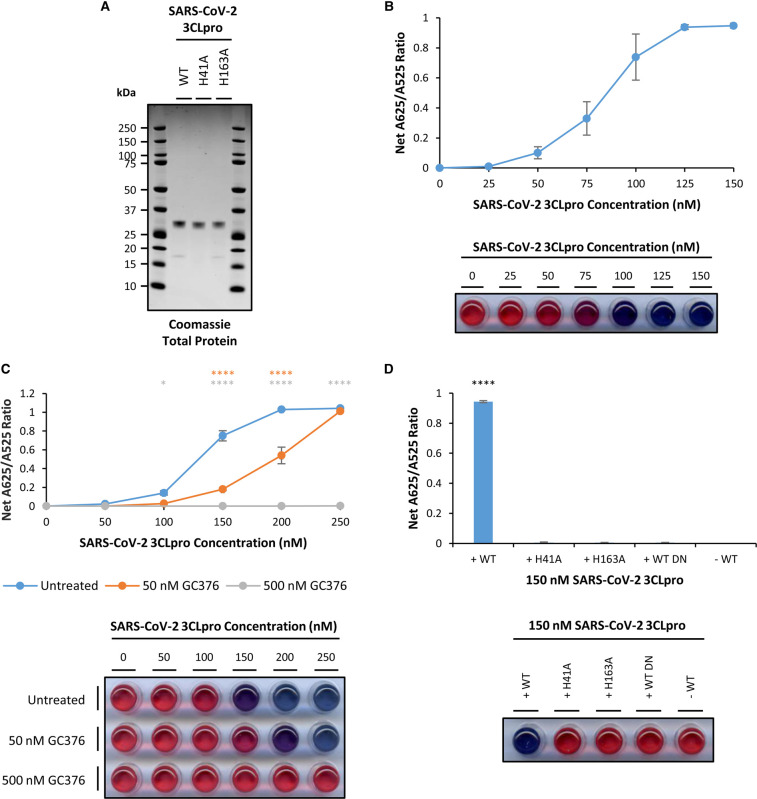
Recombinant SARS-CoV-2 3CLpro shows a concentration-dependent increase in net A625/A525 ratio which is dependent upon 3CLpro activity. (A) 1 µg untagged, purified recombinant SARS-CoV-2 wild-type (WT), H41A and H163A 3CLpro protein was separated by SDS–PAGE and total protein visualised with Coomassie Blue stain; (B) Recombinant SARS-CoV-2 3CLpro shows a concentration dependent increase in net A625/A525 ratio (error bars represent means ± standard deviation; *n *= 3 independent experiments); (C) Compared with untreated (blue points) samples, treatment with 50 nM (orange points) and 500 nM (grey points) GC376 significantly reduces recombinant SARS-CoV-2 3CLpro concentration-dependent increases in net A625/A525 ratio (error bars represent means ± standard deviation; *n *= 3 independent experiments; **** *P* < 0.0001, * *P* < 0.05 by two-way ANOVA Dunnett's Multiple Comparisons Test compared with Untreated sample); (D) Recombinant SARS-CoV-2 H41A and H163A 3CLpro mutants and wild-type denatured (WT DN) recombinant SARS-CoV-2 3CLpro show no significant increase in net A625/A525 ratio above background levels (−WT) compared with wild-type (WT) SARS-CoV-2 3CLpro (error bars represent means ± standard deviation; *n *= 3 independent experiments; **** *P* < 0.0001 by one-way ANOVA Dunnett's Multiple Comparisons Test). A representative visual image from independent replicate experiments is shown in a panel below the graph.

Recombinant SARS-CoV-2 3CLpro was expressed and purified as a 48 kDa N-terminally His-tagged Smt3 domain fusion protein from *E. coli* BL21(DE3)-RIL cells by immobilised metal ion affinity chromatography (IMAC). The purified protein was then cleaved with His-tagged SUMO protease and both the His-tagged SUMO protease and the 14 kDa His-tagged Smt3-domain N-terminal portion of the cleaved fusion protein was removed by IMAC, leaving the cleaved 34 kDa C-terminal portion of the fusion protein encoding SARS-CoV-2 wild-type (WT) 3CLpro as its native amino acid sequence (Supplementary Figure S1 and Figure 1A). Reducing agents such as DTT are reported to maintain the stability of purified recombinant 3CLpro [20], but are incompatible with the downstream applications and were therefore omitted. However, a gradual reduction in the protease activity of purified recombinant 3CLpro enzymes while in storage was consequently observed throughout the course of this study and caused some variability in 3CLpro activity.

To test its protease activity, increasing concentrations of purified recombinant SARS-CoV-2 3CLpro were incubated in the presence of a native 3CLpro peptide substrate encoding the 10 mer N-terminal self-cleavage site of SARS-CoV-2 3CLpro in the linker region with a Glu_4_ stabilisation domain at the N-terminus and a Cysteine (Cys) residue at the C-terminus (H_2_N-EEEETSAVLQSGFRC-COOH) [21]. The protease reaction mix was then added to a solution of citrate-stabilised AuNPs. In the absence of 3CLpro, the peptide substrate is not cleaved and anchors to the AuNPs via the C-terminal Cys residue and stabilises the AuNPs via the N-terminal Glu_4_ domain through electrostatic repulsion resulting in a distinctive red colour and a low net A625/A525 ratio. However, in the presence of higher concentrations of 3CLpro, the peptide substrate is cleaved within the linker region, removing the stabilisation motif from the anchored peptide domain and thus allowing the AuNPs to aggregate resulting in a marked colour change to blue and an increase in the net A625/A525 ratio [19]. The results demonstrate that increasing the concentration of SARS-CoV-2 3CLpro from 50 nM to 125 nM is associated with an increase in the net A625/A525 ratio with saturation observed at the higher concentration (Figure 1B). These data are consistent with cleavage of the peptide substrate in the presence of purified recombinant SARS-CoV-2 3CLpro demonstrating robust protease activity, and provide proof-of-principle for the colorimetric 3CLpro AuNP protease assay.

To confirm that cleavage of the 3CLpro substrate peptide in the AuNP protease assay is due to the protease activity of purified recombinant 3CLpro, a potent competitive inhibitor of 3CLpro, GC376, was employed [22–24]. In the presence of 50 nM GC376, a significant reduction in net A625/A525 ratio is observed between 100 to 200 nM SARS-CoV-2 3CLpro concentration, while at 500 nM GC376 a profound reduction in net A625/A525 ratio is observed up to 250 nM SARS-CoV-2 3CLpro concentration without any observable colour change in the 3CLpro AuNP protease assay (Figure 1C). Similarly, the broad spectrum chymotrypsin inhibitor Chymostatin has been previously reported to show limited inhibition of 3CLpro enzymes [25]. In the presence of 100 µM Chymostatin, significant decreases in net A625/A525 ratio are observed at 75 to 100 nM SARS-CoV-2 3CLpro concentration (Supplementary Figure S2).

Moreover, SARS-CoV-2 3CLpro mutants with mutations at residue 41 (H41A) and residue 163 (H163A) were expressed and purified. Both mutations have previously been reported to cause catalytic inactivation in SARS-CoV 3CLpro with H41A disrupting the catalytic dyad at its active site and H163A interfering with the substrate binding domain [26]. In addition, SARS-CoV-2 WT 3CLpro was also denatured through incubation at 95°C prior to use. In the AuNP protease assay, both H41A and H163A mutants and denatured WT SARS-CoV-2 3CLpro showed no increase in net A625/A525 ratio above background levels nor any observable colour change, in contrast with wild-type SARS-CoV-2 3CLpro which showed marked and significantly increased net A625/A525 ratio at 150 nM concentration (Figure 1D).

Together, these data are consistent with cleavage of the 3CLpro substrate peptide in the 3CLpro AuNP protease assay being dependent upon the protease activity of purified recombinant SARS-CoV-2 3CLpro.

### The AuNP protease assay specifically detects 3CLpro from β-coronaviruses

To have use as a clinical diagnostic tool, the AuNP protease assay must display an acceptable level of specificity. We therefore tested the assay using two related proteases. The human coronavirus OC43 also belongs to the β-genera, and the 3CLpro it encodes shows 48% overall sequence identity with SARS-CoV-2 3CLpro, and while previous research has indicated similar substrate specificities, some differences have been observed [16]. The 3C proteases encoded by picornaviruses such as Rhinoviruses are also homologous to the coronavirus 3CLpro [14]. In particular, human Rhinovirus Type 14 (HRV-14) which is an endemic human virus also causes the common cold [27]; the 3C protease it encodes (HRV-14 3Cpro) shows no significant overall sequence similarity with SARS-CoV-2 3CLpro.

**Figure 2.**
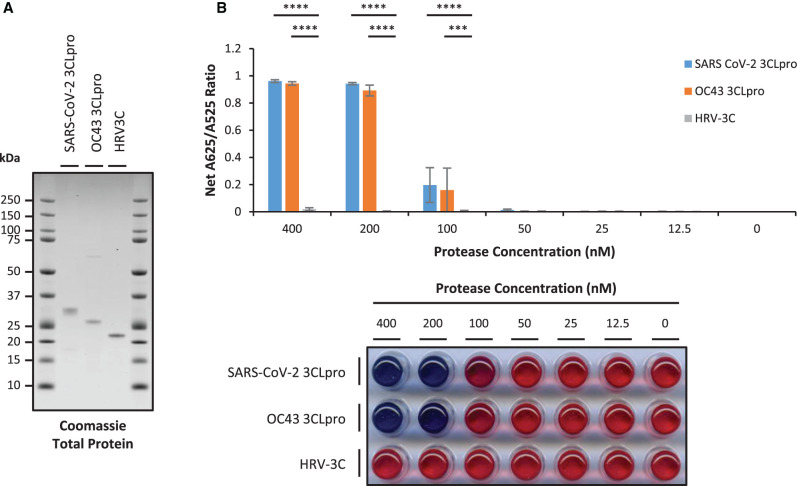
Recombinant SARS-CoV-2 and OC43 3CLpro but not HRV-3C shows a concentration-dependent increase in net A625/A525 ratio. (**A**) 1 µg purified recombinant SARS-CoV-2 and OC43 3CLpro and HRV3C protein was separated by SDS–PAGE and total protein visualised with Coomassie Blue stain; (**B**) Recombinant SARS-CoV-2 (blue bars) and OC43 (orange bars) 3CLpro show a concentration dependent increase in net A625/A525 ratio but recombinant HRV-3C (grey bars) shows no significant increase in net A625/A525 ratio above background levels (error bars represent means ± standard deviation; *n *= 3 independent experiments; **** *P* < 0.0001, *** *P* < 0.0005 by two-way ANOVA Tukey's Multiple Comparisons Test). A representative visual image from independent replicate experiments is shown in a panel below the graph.

To test the specificity of the AuNP protease assay, recombinant OC43 3CLpro was expressed and purified concomitantly with SARS-CoV-2 3CLpro as described (Supplementary Figure S3). Purified recombinant His-tagged HRV-14 3Cpro (HRV-3C) from a commercial vendor was also employed (Figure 2A). In the presence of 3CLpro substrate peptide, both SARS-CoV-2 and OC43 3CLpro show a similar profile of protease activity with net A625/A525 ratio increasing as 3CLpro concentration increases from 100 nM to 400 nM. In contrast, HRV-3C shows significantly reduced net A625/A525 ratio with no increase above background levels up to 400 nM HRV-3C (Figure 2B).

To confirm that purified recombinant SARS-CoV-2 and OC43 3CLpro and HRV-3C are all catalytically active under these conditions, His-tagged Smt3-fusion proteins containing substrate sequences for 3CLpro (TSAVLQSGFR) [21] or HRV-3C (LEVLFQGP) [28] at the C-terminus were expressed and purified from *E. coli* BL21(DE3)-RIL cells by IMAC (Supplementary Figure S4). The data show that the 3CLpro substrate protein remains uncleaved as the 17 kDa full-length fusion protein in the presence of 400 nM HRV-3C, but is cleaved in the presence of either 400 nM SARS-CoV-2 or 400 nM OC43 3CLpro resulting in a slight shift to the 15.5 kDa cleaved size and loss of the C-terminal His-tag as detected by Western blot. In contrast, the HRV-3C substrate protein remains uncleaved as the 17 kDa full-length fusion protein in the presence of either 400 nM SARS-CoV-2 or 400 nM OC43 3CLpro, but is cleaved in the presence of 400 nM HRV-3C to cause a slight shift to the 15.5 kDa cleaved size and loss of the C-terminal His-tag as detected by Western blot (Supplementary Figure S5).

These results indicate that while SARS-CoV-2 and OC43 3CLpro can cleave the 3CLpro substrate peptide and elicit a positive result in the 3CLpro AuNP protease assay, HRV-3C protease is not able to cleave the 3CLpro substrate peptide under these conditions despite being catalytically active.

### Inclusion of catalytic hybrid dimers and optimised peptide design improves the specific sensitivity of the AuNP protease assay

While an assay that specifically detects 3CLpro from β-coronavirus species could have use in clinical diagnostics, it was important to improve specific sensitivity towards SARS-CoV-2 3CLpro to facilitate more accurate diagnosis of CoVID-19 disease. To this end, two independent approaches were investigated.

**Figure 3.**
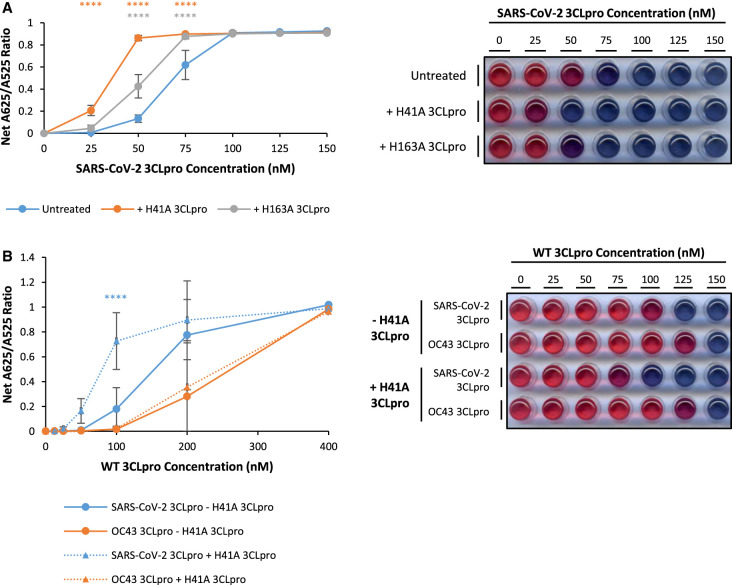
Addition of recombinant SARS-CoV-2 H41A 3CLpro mutant protein significantly enhances sensitivity and specificity for SARS-CoV-2 3CLpro with the AuNP protease assay. (**A**) Addition of 750 nM SARS-CoV-2 H41A (orange points) and H163A (grey points) 3CLpro mutant proteins significantly increases net A625/A525 ratio compared with untreated (blue points) protease reactions with SARS-CoV-2 3CLpro (error bars represent means ± standard deviation; *n *= 3 independent experiments; **** *P* < 0.0001 by two-way ANOVA Dunnett's Multiple Comparisons Test compared with Untreated sample); (**B**) Addition of 750 nM SARS-CoV-2 H41A 3CLpro mutant protein (triangular points and dashed lines) significantly increases net A625/A525 ratio compared with untreated (circular points and solid lines) protease reactions with SARS-CoV-2 3CLpro (blue points) but not with OC43 3CLpro (orange points) (error bars represent means ± standard deviation; *n *= 4 independent experiments; **** *P* < 0.0001 by two-way ANOVA Tukey's Multiple Comparisons Test compared with — H41A 3CLpro sample). A representative visual image from independent replicate experiments is shown in a panel beside the graph.

It has previously been shown that SARS-CoV 3CLpro is only catalytically active as an asymmetric dimer in which only one protomer is active, while monomers are catalytically inactive [29]. Consequently, addition of inactive mutant SARS-CoV 3CLpro monomers into hybrid dimers increases the enzymatic activity of wild-type SARS-CoV 3CLpro [29]. To determine whether this is also the case for SARS-CoV-2 3CLpro in the AuNP protease assay, increasing concentrations of SARS-CoV-2 WT 3CLpro were supplemented with 750 nM inactive mutant SARS-CoV-2 H41A or H163A 3CLpro in the presence of native 3CLpro substrate peptide. The data show that addition of either mutant significantly increases the net A625/A525 ratio above the untreated sample at 25 nM to 75 nM WT 3CLpro, and this increase appears consistently more pronounced for the H41A mutant than H163A (Figure 3A).

Although 3CLpro from different groups of coronaviruses are structurally conserved there are distinctions [14,16]. We therefore hypothesised that inactive mutant SARS-CoV-2 3CLpro monomers would stabilise hybrid dimers with SARS-CoV-2 3CLpro, but not OC43 3CLpro monomers, and would therefore specifically increase enzymatic activity of SARS-CoV-2 3CLpro in the AuNP protease assay. To test this notion, purified recombinant SARS-CoV-2 or OC43 WT 3CLpro was incubated with 3CLpro substrate peptide in the presence or absence of 750 nM SARS-CoV-2 H41A 3CLpro. The AuNP protease assays show that net A625/A525 ratio is significantly increased at 100 nM SARS-CoV-2 WT 3CLpro in the presence of 750 nM SARS-CoV-2 H41A 3CLpro, but is unaffected for OC43 WT 3CLpro (Figure 3B). Thus, addition of inactive SARS-CoV-2 H41A 3CLpro specifically enhances the sensitivity of the AuNP protease assay for SARS-CoV-2 3CLpro.

**Figure 4.**
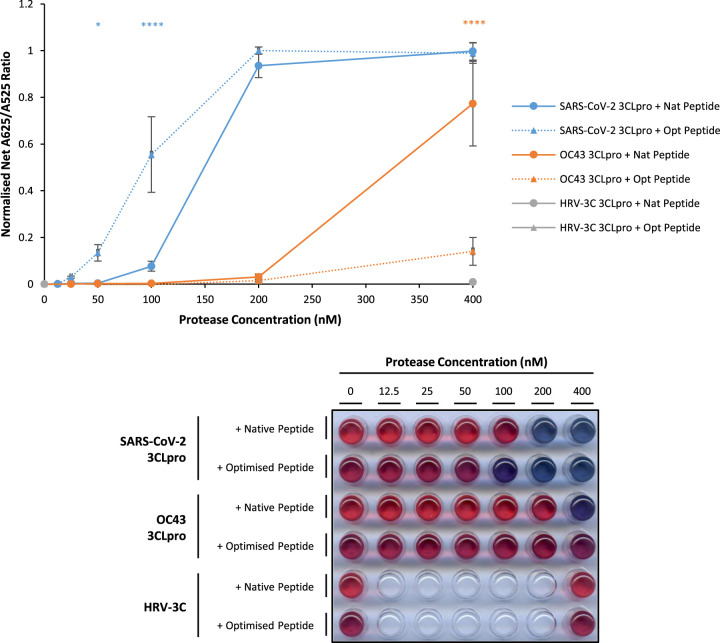
Optimised peptide significantly enhances sensitivity and specificity for SARS-CoV-2 3CLpro. In the presence of optimised (Opt) peptide (triangular points and dashed line) compared with native (Nat) peptide (circular points and solid line), normalised net A625/A525 ratio is increased for SARS-CoV-2 3CLpro (blue points) and decreased for OC43 3CLP (orange points), and not significantly increased above background levels for HRV-3C (grey points) (error bars represent means ± standard deviation; *n *= 3 independent experiments; **** *P* < 0.0001, * *P* < 0.05 by two-way ANOVA Tukey's Multiple Comparisons Test compared with + Nat Peptide sample). A representative visual image from independent replicate experiments is shown in a panel below the graph.

Previous research has shown that although 3CLpro from different coronavirus species show many similarities in substrate specificities, there are differences in substrate preference [16]. These data were used to design an optimised 3CLpro substrate peptide to maximise specific activity for the highly conserved SARS-CoV 3CLpro over 3CLpro from other coronavirus groups (H_2_N-EEEETHVVLQASFRC-COOH). In the AuNP protease assay, background A625/A525 ratio levels are higher for the optimised 3CLpro substrate peptide than the native 3CLpro substrate peptide (which reflects differences in the purification process). Nevertheless, normalised net A625/A525 ratio is significantly increased for 50–100 nM SARS-CoV-2 3CLpro, in the presence of optimised compared with native 3CLpro substrate peptide. In contrast, normalised net A625/A525 ratio is decreased for 400 nM OC43 3CLpro in the presence of optimised compared with native 3CLpro substrate peptide (Figure 4). These results demonstrate that the optimised 3CLpro substrate peptide enhances the specificity and sensitivity of the AuNP protease assay compared with the native 3CLpro substrate peptide.

The reported *K*_M_ value of SARS-CoV-2 3CLpro for a similar peptide substrate to the native 3CLpro peptide substrate is 245 µM [30]. Since the concentration of 3CLpro substrate peptide used in the 3CLpro AuNP protease assay is 100 µM, increasing the concentration of substrate peptide should increase the sensitivity of detection. Therefore, increasing concentrations of native 3CLpro substrate peptide were incubated with 150 nM SARS-CoV-2 3CLpro over the time course shown. As expected, increasing the concentration of substrate peptide significantly increases the rate of reaction for SARS-CoV-2 3CLpro with 400 µM and 200 µM substrate peptide (Supplementary Figure S6) and effectively improves the sensitivity of detection within 90 min using the AuNP protease assay (Supplementary Figure S7).

### The AuNP protease assay is effective at room temperature

Although a diagnostic assay with a 37°C incubation is feasible for ‘in the field’ application using a heat block or water bath, incubation at room temperature would be more amenable and advantageous as it would not require any additional equipment.

To characterise the sensitivity of detection within 90 min using the AuNP assay, increasing concentrations of SARS-CoV-2 3CLpro were incubated with 100 µM native 3CLpro substrate peptide across a temperature range. The data show that net A625/A525 ratio significantly increases as temperature of incubation increases from 23°C and 30°C to 37°C at 100 nM SARS-CoV-2 3CLpro and from 23°C to 37°C at 150 nM SARS-CoV-2 3CLpro. However, increasing the temperature of incubation from 37°C to 44°C significantly decreases the net A625/A525 at 100 to 250 nM SARS-CoV-2 3CLpro (Supplementary Figure S8).

**Figure 5.**
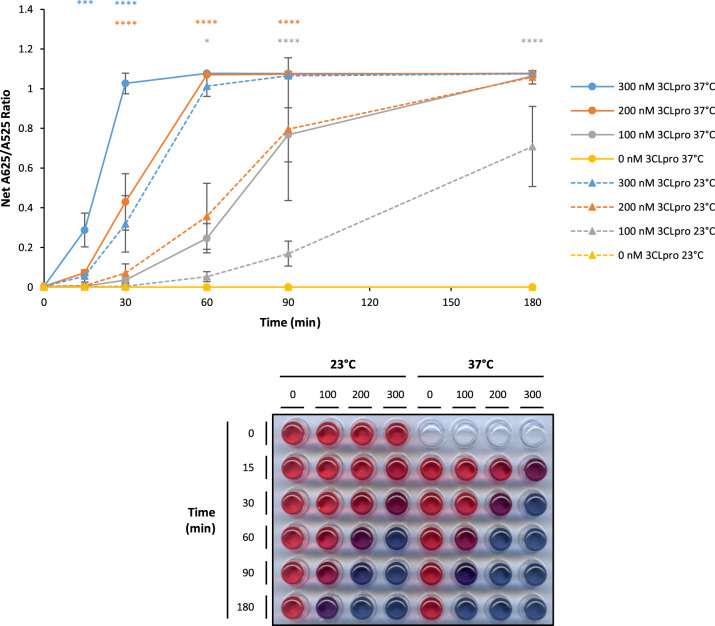
SARS-CoV-2 3CLpro is active at 23°C but maximal at 37°C. SARS-CoV-2 3CLpro protease reactions incubated at 23°C (triangular points and dashed lines) show increases in net A625/A525 at 300, 200 and 100 nM SARS-CoV-2 3CLpro but the rate of increase in net A625/A525 ratio is reduced compared with incubation at 37°C (circular points and solid lines) (error bars represent means ± standard deviation; *n *= 4 independent experiments; * *P* < 0.05, *** *P* < 0.001, **** *P* < 0.0001 by two-way ANOVA Tukey's Multiple Comparisons Test). A representative visual image from independent replicate experiments is shown in a panel below the graph.

To investigate this further, increasing concentrations of SARS-CoV-2 3CLpro were incubated with 100 µM native 3CLpro substrate peptide at 23°C or 37°C for up to 180 min. The results show that the rate of increase in net A625/A525 is significantly improved at 37°C compared with 23°C at 100, 200 and 300 nM SARS-CoV-2 3CLpro. Despite this, with 300 nM SARS-CoV-2 3CLpro at 23°C, maximal net A625/A525 is still achieved within 90 min and a visually obvious colour change is observed within 30 min. With 200 nM SARS-CoV-2 3CLpro at 23°C, maximal net A625/A525 is still reached within 180 min and a visually obvious colour change is seen within 60 min. Moreover, even with 100 nM SARS-CoV-2 3CLpro at 23°C, there are marked and reproducible increases in net A625/A525 compared with the 0 nM SARS-CoV-2 3CLpro sample from 60 min and a visually obvious colour change between 90 and 180 min (Figure 5).

Therefore, within the range of temperatures tested, 37°C is optimal to maximise SARS-CoV-2 3CLpro activity against the native 3CLpro substrate peptide as expected [26,31]. However, the protease reaction can still proceed at 23°C within acceptable time frames under these conditions which makes the assay amenable for use outside of a clinical laboratory. Moreover, reaction rates can be further enhanced by the use of hybrid dimerisation with inactive mutant SARS-CoV-2 3CLpro protomers, use of optimised substrate peptides and by increasing the concentration of substrate peptide as described above.

### The AuNP protease assay detects SARS-CoV-2 3CLpro activity in transfected human cell lysates

**Figure 6.**
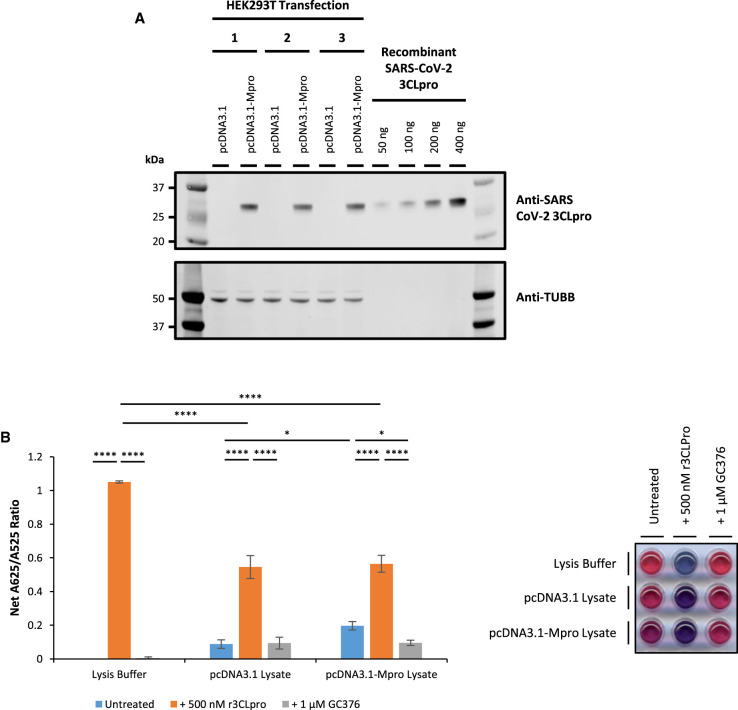
Detection of cellular SARS-CoV-2 3CLpro expression and activity in pcDNA3.1-Mpro transfected HEK293T cell lysates. (**A**) Cellular SARS-CoV-2 3CLpro protein is exogenously expressed in HEK293T cells transfected with pcDNA3.1-Mpro plasmid. Proteins were separated by SDS–PAGE and visualised by Western blot. (**B**) Addition of untreated lysate (blue bars) from pcDNA3.1-Mpro (3CLpro expression vector) transfected HEK293T cells or 500 nM recombinant SARS-CoV-2 3CLpro (r3CLpro; orange bars) significantly increases net A625/A525 ratio compared with untreated lysate from pcDNA3.1 (control vector) transfected HEK293T cells or lysate treated with 1 µM GC376 (grey bars). Note: For Lysis Buffer + 1 µM GC376 samples, 500 nM r3CLpro was also added (error bars represent means ± standard deviation; *n *= 3 independent experiments; * *P* < 0.05, **** *P* < 0.0001 by two-way ANOVA Tukey's Multiple Comparisons Test). A representative visual image from independent replicate experiments is shown in a panel beside the graph.

To establish whether the AuNP protease assay is able to detect SARS-CoV-2 3CLpro activity in the context of a human cell, human embryonic kidney cells (HEK293T) were transfected with pcDNA3.1-Mpro [32], resulting in strong expression of SARS-CoV-2 3CLpro in cell lysates (Figure 6A).

Equal concentrations of cell lysates from HEK293T cells transfected with pcDNA3.1 (control vector) and pcDNA3.1-Mpro (3CLpro expression vector) were incubated with 400 µM 3CLpro peptide substrate in the presence of 750 nM recombinant SARS-CoV-2 H41A 3CLpro mutant protein for 90 min and analysed with the AuNP protease assay by adding 15 µl digested peptide solution to 185 µl citrate-stabilised AuNP solution.

In the presence of control vector, there is no significant increase in net A625/A525 ratio and no effect of treatment with GC376 3CLpro inhibitor (Figure 6B). When 500 nM recombinant SARS-CoV-2 3CLpro is added to the reaction, there is a significant decrease in net A625/A525 ratio in the presence of HEK293T cell lysates compared with the lysis buffer control, suggesting that components of the cell lysate may interfere with the AuNP protease assay (Figure 6B, orange bars).

Importantly, however, there is a modest but significant (2.3-fold; *P* < 0.05) increase in net A625/A525 ratio in the samples transfected with pcDNA3.1-Mpro, compared with control vector (Figure 6B, blue bars). Moreover, addition of 1 µM GC376 3CLpro inhibitor to the protease reaction significantly decreases the net A625/A525 ratio in samples transfected with pcDNA3.1-Mpro, down to background levels comparable to samples transfected with control vector (Figure 6B; compare blue and grey bars). This is consistent with the change in net A625/A525 ratio being due to the protease activity of the exogenously expressed SARS-CoV-2 3CLpro in the cell lysates.

Together, these results demonstrate that protease activity of SARS-CoV-2 3CLpro exogenously expressed in HEK293T cells can be reproducibly detected in cell lysates using the AuNP protease assay. However, interference from components of the cell lysate limits the dynamic range of the AuNP protease assay and thus impedes the limit of detection and prevents saturation of the assay.

It is likely that the major source of interference derives from a protein component of the cell lysate non-specifically obstructing the cleaved peptide substrate from interacting with the AuNPs, rather than an inhibition of the protease reaction *per se*. Therefore, to overcome this issue cleaved peptide substrate was enriched from the post-reaction sample by size-exclusion chromatography. This was achieved by incubating equal concentrations of cell lysates from HEK293T cells transfected with pcDNA3.1 (control vector) and 0.5 µg (low expression) or 2.5 µg (high expression) pcDNA3.1-Mpro (3CLpro expression vector) with 150 µM 3CLpro peptide substrate in the presence of 750 nM recombinant SARS-CoV-2 H41A 3CLpro mutant protein for 90 min. The post-reaction sample (Input) was then subject to size exclusion centrifugal filtration with a 10 kDa cut-off to enrich the peptide substrate in the flow-through (10K FT). The samples were then analysed with the AuNP protease assay by adding 15 µl Input and 10 µl 10K FT sample to 185 µl and 190 µl citrate-stabilised AuNP solution, respectively.

**Figure 7.**
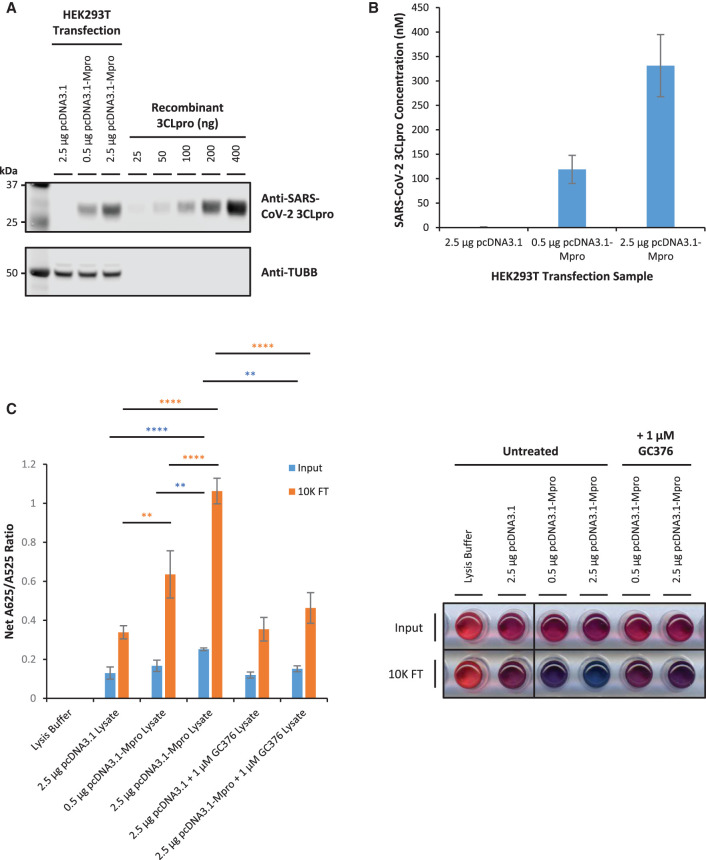
Post-reaction enrichment of peptide substrate enables detection of cellular SARS-CoV-2 3CLpro activity in pcDNA3.1-Mpro-transfected HEK293T cell lysates at high and low expression levels. (**A**) Cellular SARS-CoV-2 3CLpro protein is exogenously expressed at low and high levels in HEK293T cells transfected with 0.5 µg and 2.5 µg pcDNA3.1-Mpro plasmid (3CLpro expression vector), respectively. Proteins were separated by SDS–PAGE and visualised by Western blot. (**B**) Quantification of 3CLpro concentration in transfected HEK293T cell lysate protease reactions by densitometric analysis of Western blots by calibration against a five-point dilution range of recombinant SARS-CoV-2 3CLpro (error bars represent means ± standard deviation; *n* = 3 independent experiments). (**C**) Addition of untreated lysate from pcDNA3.1-Mpro-transfected HEK293T cells significantly increases net A625/A525 ratio compared with untreated lysate from pcDNA3.1 (control vector)-transfected HEK293T cells or lysate treated with 1 µM GC376 for 0.5 µg and 2.5 µg pcDNA3.1-Mpro plasmid in 10K FT samples (orange bars) but only for 2.5 µg pcDNA3.1-Mpro plasmid in Input samples (blue bars) (error bars represent means ± standard deviation; *n* = 3 independent experiments; ** *P* < 0.01, **** *P* < 0.0001 by one-way ANOVA Tukey's Multiple Comparisons Test). A representative visual image from independent replicate experiments is shown in a panel beside the graph.

Human HEK293T cells transfected with 0.5 µg and 2.5 µg pcDNA3.1-Mpro results in low and high levels of SARS-CoV-2 3CLpro expression, respectively (Figure 7A). Quantification of 3CLpro concentration in transfected HEK293T cell lysates by densitometric analysis of Western blots indicates that the mean molar concentration of cellular SARS-CoV-2 3CLpro present in the protease reactions for 0.5 µg and 2.5 µg pcDNA3.1-Mpro samples is 119 nM and 331 nM, respectively (Figure 7B).

For the Input samples, compared with control vector, there is a modest but significant increase in net A625/A525 in the samples transfected with 2.5 µg pcDNA3.1-Mpro (2.0-fold; *P* < 0.0001), but not 0.5 µg pcDNA3.1-Mpro, which is significantly reduced in the presence of 1 µM GC376 3CLpro inhibitor consistent with previous results (Figure 7C; compare blue bars). However, for 10K FT samples in which peptide substrate has been enriched from the post-reaction sample through size exclusion centrifugal filtration, there are significant increases in net A625/A525 in samples transfected with 0.5 µg (1.9-fold; *P* < 0.01) and 2.5 µg pcDNA3.1-Mpro (3.1-fold; *P* < 0.0001) compared with control vector, eliciting a visually obvious colour change to purple and blue, respectively (Figure 7C; compare orange bars and see panel lower row). Moreover, addition of 1 µM GC376 3CLpro inhibitor significantly decreases the net A625/A525 ratio in samples transfected with 2.5 µg pcDNA3.1-Mpro, consistent with the increase in net A625/A525 being due to the protease activity of the exogenously expressed SARS-CoV-2 3CLpro in the cell lysates (Figure 7C; compare orange bars).

Taken together, these results show that the AuNP protease assay can reproducibly detect specific protease activity from cellular SARS-CoV-2 3CLpro exogenously expressed in HEK293T cells and that interference from components of the cell lysate can be overcome by enrichment of the peptide substrate from the post-reaction sample resulting in a robust and significant increase in net A625/A525 and a visually apparent colour change with the AuNP protease assay in the presence of cellular SARS-CoV-2 3CLpro.

## Discussion

In this study, an *in vitro* colorimetric AuNP protease assay is adapted to specifically detect the protease activity of β-genera coronavirus 3CLpro enzymes. The data show specific detection of purified recombinant 3CLpro from SARS-CoV-2 and OC43 human coronaviruses, providing a clear red to blue colour change, which can be easily interpreted by eye and which can be quantified by the A625/A525 ratio. There is no colour change with the homologous HRV-14 3C protease under these conditions (Figure 2B). The study also demonstrates that increasing the substrate peptide concentration up to 400 µM increases the sensitivity and speed of the assay, with positive results obtained within 15 min of incubation at 37°C (Supplementary Figures S6, S7). However, the concentration of the substrate peptide used will be limited practically by its solubility and cost. The assay is also compatible with incubation at room temperature and although, as expected, there is a reduction in the rate of protease reaction, a distinct colour change can be obtained within 30 min of incubation with 100 µM substrate peptide and 300 nM SARS-CoV-2 3CLpro (Figure 6). Therefore, with future development, this assay is compatible with incubation at room temperature or gentle warming, e.g. using simple laboratory equipment such as a water bath.

This study demonstrates that the AuNP protease assay can be used to detect cellular SARS-CoV-2 3CLpro activity in transfected HEK293T cells *in vitro*. However, although the increase in net A625/A525 ratio is consistent and significant, it is modest and the colour change is slight due to interference from components of the cell lysate (Figure 6). The major source of this interference derives from protein components of the cell lysate non-specifically obstructing interactions between the cleaved peptide substrate and the AuNPs, rather than inhibition of the protease reaction. Consequently, size exclusion centrifugal filtration is shown to be a successful experimental strategy to enrich the peptide substrate from the post-reaction sample to overcome the interference from the cell lysate. This enables the AuNP protease assay to detect cellular SARS-CoV-2 3CLpro activity in transfected HEK293T cells *in vitro* when SARS-CoV-2 3CLpro is expressed at low and high levels, with robust and significant increases in net A625/A525 and visually obvious colour changes to purple and blue, respectively (Figure 7). Although this strategy would not be appropriate for a point-of-care diagnostic assay, it demonstrates potential for further development of the AuNP protease assay as a diagnostic test which would be necessary. Furthermore, components of the cell lysate increases background A625/A525 ratio levels in the absence of SARS-CoV-2 3CLpro expression which limits the dynamic range of the AuNP protease assay and remains to be fully resolved (Figure 7). A strategy in which SARS-CoV-2 3CLpro is enriched from a lysate sample prior to the protease reaction may therefore be preferable for future development of the assay.

Intracellular 3CLpro activity has previously been detected in SARS-CoV-2-infected human cell lines [32,33], but it remains to be determined whether the *in vitro* AuNP protease assay is sufficiently sensitive to detect cellular 3CLpro activity from SARS-CoV-2 infected human cell line lysates. Protein levels of 3CLpro appear to be 50-fold lower in the context of viral infection, compared with transient transfection of the protease as an exogenous transgene (Supplementary Figure S9). This corresponds to a protease reaction concentration of <10 nM, just below the 25 nM limit of detection demonstrated for recombinant SARS-CoV-2 3CLpro in this study (Figure 3A). Thus, although detection of SARS-CoV-2 3CLpro activity in SARS-CoV-2-infected cell line lysates with the AuNP protease assay has not yet been successful, with further optimisation and development this would likely be achievable.

3CLpro is a non-structural protein so it is not likely to be released in virions, but is thought to reside in double-membrane vesicles associated with the endoplasmic reticulum in infected cells [34,35]. Despite this, CoVID-19 patients are reported to produce high titre antibody responses to 3CLpro which is possibly released from infected cells at the end of the viral life cycle [36]. Detection of SARS-CoV-2 3CLpro activity and protein expression by the AuNP assay and Western blot in nasopharyngeal/oropharyngeal swab and saliva samples from a small cohort of CoVID-19 patients (*n* = 5) has not been successful and was limited by a low yield of cellular protein (data not shown). Further research will be required to demonstrate whether the AuNP protease assay is capable of detecting 3CLpro activity in SARS-CoV-2-infected patient samples, and indeed which specimen types show greatest levels of 3CLpro activity. Since 3CLpro is expressed and active at an early stage in the coronavirus infection cycle it is potentially possible that a sufficiently sensitive assay to detect 3CLpro activity could detect SARS-CoV-2 infection at an earlier stage of infection than other diagnostic tests, which depend predominantly upon antigenic proteins and RNA contained within virion particles, possibly allowing identification of infected patients prior to transmission and earlier treatment of at-risk individuals [13]. In this regard, laboratory-based approaches to detect 3CLpro activity such as fluorescent reporter substrates may offer superior sensitivity however the limit of detection is more likely limited by the dimer-to-monomer dissociation of the 3CLpro enzyme [20].

Since a number of human coronaviruses are already endemic in the global population [13], it would be advantageous for a clinical diagnostic test to specifically distinguish SARS-CoV-2 from other coronavirus infections. In this study, two independent strategies are demonstrated to improve specific sensitivity for SARS-CoV-2 3CLpro compared with OC43 3CLpro: hybrid dimerisation with inactive mutant SARS-CoV-2 3CLpro protomers (Figure 3B), and use of an optimised substrate peptide (Figure 4). Indeed, it is possible that future work could further optimise the substrate peptide to improve the specific activity for SARS-CoV-2 3CLpro including the length of the substrate sequence within the peptide and inclusion of more optimal amino acids which could include modified or non-canonical amino acids as indicated by previous studies [16,37]. Unfortunately, the optimised substrate peptide used in this study was not compatible with cell lysate samples, but this is likely related to the specific sequence of the peptide and could be overcome by adapting the sequence appropriately. In addition, past research has highlighted the role of 3D domain swapping in stabilising 3CLpro in its active conformation [38]. Further research may also elucidate new mutants that favour forming hybrid dimers in which the mutant protomer is in the inactive position and other strategies to enhance 3CLpro activity and the sensitivity of its detection in this assay.

The results from this study indicate that the AuNP protease assay is likely to detect 3CLpro from at least the beta-genera of coronaviruses (β-CoV) which includes human coronaviruses OC43 and HKU1 (lineage A), SARS-CoV and CoV-2 (lineage B) and MERS (lineage C). While SARS-CoV and MERS are not thought to be widespread among the human population and are currently unlikely to be encountered in the clinical diagnostic setting, OC43 and HKU1 are considered endemic [13]. Although SARS-CoV and CoV-2 3CLpro show a high degree of similarity (96% identity), the overall sequence conservation for MERS (51%), OC43 (48%) and HKU1 (49%) 3CLpro is much lower and thus it is realistic that the strategies identified to improve the specific sensitivity of the AuNP protease assay for SARS-CoV-2 will be effective against non-lineage B β-CoV, although this remains to be shown experimentally. Despite this, if this assay was to be used in the arena of clinical diagnosis, it may be judicious to validate positive results using a gold-standard diagnostic assay such as the RT-PCR test where available to identify false positive results caused by coronavirus infection other than SARS-CoV-2 and to quantify the viral titre [39]. Importantly, the AuNP protease assay should also detect 3CLpro from all known variants of SARS-CoV-2 as the amino acid sequence of this enzyme is highly conserved and no mutations have yet been observed on the 3CLpro encoding section of the ORF1ab polyprotein [40]. However, the assay could also be easily adapted through modification of peptide substrate sequence to target detection of 3CLpro from other coronaviruses that are already known or which may arise in the future, as well as proteases encoded by other viruses with specific substrate preferences and other diseases conditions in which such proteases are diagnostically relevant [19].

Thus, with further appropriate development, the AuNP protease assay for detection of SARS-CoV-2 3CLpro could occupy a position in the clinical setting as a rapid and cost effective point of care diagnostic assay for the identification of suspected SARS-CoV-2-infected patients who could then be referred for further diagnostic tests, quarantine and care to relieve the burden on diagnostic testing facilities. The benefits of this assay include cost effectiveness (the cost per test in this small scale study is <£0.10 per assay) without the need for complex equipment or highly trained staff to process and analyse samples, rapid results in the form of a colour change which can be easily interpreted by a lay person, and high versatility potentially enabling adaptation to other viruses currently in circulation or which could arise in the future.

## Materials and methods

### Cloning of 3CLpro

Codon-optimised double-stranded DNA fragments encoding Nsp5 of OC43 (OC43 3CLP Insert) and SARS-CoV-2 (CoV2 3CLP Insert) replicase polyprotein 1a for expression in *E. coli* were synthesised (IDT) (Table 1) and amplified by PCR using HCoV OC43 3CL c1F and HCoV 3CLP c1R primers and SARS CoV2 3CL c3F and c3R primers, respectively (Table 1). The PCR products were digested with BamHI and XhoI and ligated with BamHI/XhoI-digested pET28-His_10_Smt3 DNA (donated by Prof. Fu, University of Rochester). The resulting pET28-OC43 and CoV2-3CLP plasmids encode 48 kDa OC43 and SARS-CoV-2 3CLpro fusion proteins with an N-terminal His_10_Smt3 tag.

**Table 1:** A list of DNA oligonucleotides used in this study

Primer name	DNA Oligonucleotide Sequence
HCoV OC43 3CL c1F	ATTAATGGATCCGGCATTGTGAAAATGGTTAA
HCoV 3CLP c1R	CTTAAGCTCGAGTCACTGCA
SARS CoV2 3CL c3F	ATTGGTGGATCCGGTTTTCGTAAAATGGCATT
SARS CoV2 3CL c3R	CTTAAGCTCGAGTCACTGAAAGGTAACACCGCTAC
OC43 3CLP insert DNA fragment	CATATGGGATCCATGCACCACCACCACCACCACGGTGGTAGCGGCATTGTGAAAATGGTTAATCCGACCAGCAAAG TTGAACCGTGTGTTGTTAGCGTTACCTATGGTAATATGACCCTGAATGGTCTGTGGCTGGATGATAAAGTTTAT TGTCCGCGTCATGTTATTTGTAGCGCAAGCGATATGACCAATCCGGACTATACCAATCTGCTGTGTCGTGTTA CCAGCAGCGATTTTACCGTTCTGTTTGATCGTCTGAGCCTGACCGTTATGAGCTATCAGATGCGTGGTTGTAT GCTGGTTCTGACAGTTACCCTGCAGAATAGTCGTACCCCGAAATATACCTTTGGTGTTGTTAAACCGGGTGAA ACCTTTACCGTGCTGGCAGCATATAATGGTAAACCGCAGGGTGCATTTCATGTTACCATGCGTAGCAGCTATAC CATTAAAGGTAGCTTTCTGTGTGGTAGCTGCGGTAGCGTTGGTTATGTTATTATGGGTGATTGCGTGAAGTTCG TGTATATGCATCAGCTGGAACTGAGCACCGGTTGTCATACCGGCACCGATTTTAATGGTGATTTTTATGGTCCGT ATAAAGATGCCCAGGTTGTTCAGCTGCCGATTCAGGATTATATTCAGAGCGTTAATTTTCTGGCATGGCTGTATG CAGCGATTCTGAATAATTGCAACTGGTTTATCCAGAGCGATAAATGCAGCGTGGAAGATTTTAACGTTTGGGCA CTGAGCAATGGTTTTAGCCAGGTTAAAAGCGATCTGGTTATTGATGCACTGGCAAGCATGACCGGTGTTAGCC TGGAAACCCTGTTAGCAGCAATTAAACGTCTGAAAAATGGTTTTCAGGGTCGCCAGATTATGGGTAGCTGTAGC TTTGAAGATGAACTGACCCCGAGTGATGTTTATCAGCAGCTGGCAGGTATTAAACTGCAGTGACTCGAGCTTAAG
CoV2 3CLP Insert DNA Fragment	CATATGGGATCCATGAGCGGTTTTCGTAAAATGGCATTTCCGAGCGGTAAAGTTGAAGGTTGTATGGTTCAGG TTACCTGTGGCACCACCACACTGAATGGTCTGTGGCTGGATGATGTTGTTTATTGTCCGCGTCATGTTATTTGT ACCAGCGAAGATATGCTGAACCCGAATTATGAAGATCTGCTGATTCGCAAAAGCAACCATAATTTTCTGGTTCA GGCAGGTAATGTTCAGCTGCGTGTTATTGGTCATAGCATGCAGAATTGTGTGCTGAAACTGAAAGTTGATACCG CCAATCCGAAAACGCCGAAATATAAGTTTGTTCGTATTCAGCCTGGTCAGACCTTTAGCGTTCTGGCATGTTATA ATGGTAGCCCGAGCGGTGTTTATCAGTGTGCAATGCGTCCGAATTTTACCATTAAAGGCAGCTTTCTGAATGGT AGCTGTGGTAGCGTTGGTTTCAACATTGATTATGATTGCGTGAGCTTCTGCTATATGCATCATATGGAACTGCCG ACCGGTGTTCATGCAGGCACCGATCTGGAAGGTAACTTTTATGGTCCGTTTGTTGATCGTCAGACCGCACAGGC AGCAGGTACAGATACCACCATTACCGTTAATGTTCTGGCCTGGCTGTATGCAGCAGTTATTAATGGTGATCGCTG GTTTCTGAATCGTTTTACAACAACCCTGAACGATTTTAATCTGGTGGCCATGAAATATAACTATGAACCGCTGACA CAGGATCATGTTGATATTCTGGGTCCGCTGAGCGCACAGACCGGTATTGCAGTTCTGGATATGTGTGCAAGCCT GAAAGAACTGTTACAGAATGGTATGAATGGTCGTACAATTCTGGGTAGCGCACTGCTGGAAGATGAATTCACCC CGTTTGATGTTGTGCGTCAGTGTAGCGGTGTTACCTTTCAGCTCGAGCTTAAG
H41A QCL1 F	CGCTGGTACAAATAACAGCACGCGGACAATAAACAACATCATC
H41A QCL1 R	GATGATGTTGTTTATTGTCCGCGTGCTGTTATTTGTACCAGCG
H163A QCL1 F	GGTCGGCAGTTCCATATGAGCCATATAGCAGAAGCTCACG
H163A QCL1 R	CGTGAGCTTCTGCTATATGGCTCATATGGAACTGCCGACC
3CL-Sub-His F	GATCCATGGAAGAAGAAGAAACCAGCGCAGTTCTGCAGAGCGGTTTTCGTTGTC
3CL-Sub-His R	TCGAGACAACGAAAACCGCTCTGCAGAACTGCGCTGGTTTCTTCTTCTTCCATG
HRV3C-Sub-His F	GATCCATGGAAGAAGAAGAACTGGAAGTTCTGTTTCAGGGTCCGTGTC
HRV3C-Sub-His R	TCGAGACACGGACCCTGAAACAGAACTTCCAGTTCTTCTTCTTCCATG

### Site directed mutagenesis of SARS-CoV-2 3CLpro

The H41A and H163A mutants of SARS-CoV-2 3CLpro were prepared using the QuikChange Lightning Site-Directed Mutagenesis Kit (Stratagene) using H41A and H163A QCL1 F and R primers (Table 1) and pET28-CoV2-3CLP as a template. The mutations in the resulting pET28-CoV2-H41A and H163A-3CLP plasmids were verified by nucleotide sequencing.

### Expression and purification of 3CLpro

BL21-CodonPlus (DE3)-RIPL *E. coli* competent cells (Agilent) were transformed with pET28-OC43, CoV2-WT (wild-type), H41A and H163A-3CLP DNA. Cultures were grown at 30°C in 30 ml LB medium containing 30 µg/ml Chloramphenicol until OD_600_ reached 0.6 and then induced with 0.8 mM Isopropyl β-d-1-thiogalactopyranoside (IPTG) at 30°C for 3 h. The cells were harvested at 4000 rpm for 5 min and the pelleted cells were frozen at −20°C.

Frozen cell pellets were thawed on ice and resuspended in NP Buffer (50 mM NaH_2_PO_4_ pH 8.0, 300 mM NaCl) with 0.1% Triton X-100, 200 µg/ml Lysozyme and 25 U/ml Benzonase and incubated on ice for 30 min. Imidazole was then added to a final concentration of 10 mM and the cell lysates were separated at 17 000***g*** for 30 min. The cell lysates were applied to nickel-nitriloacetic acid (Ni-NTA) spin columns (Qiagen) equilibrated with 0.6 ml NP Buffer with 10 mM Imidazole. After two washes with 0.6 ml NP Buffer with 20 mM Imidazole, the His_10_Smt3-tagged 3CLpro proteins were eluted with 0.4 ml NP Buffer with 500 mM Imidazole.

The His_10_Smt3-tagged 3CLpro protein eluates were subjected to buffer exchange into NP Buffer using Amicon Ultra 30K Devices (Millipore). Samples were then supplemented with 0.5 mM DTT and incubated with 50 U His-tagged recombinant SUMO protease (Sigma) for 16 h at 4°C to remove the His_10_Smt3-tag. The His-tagged recombinant SUMO protease and cleaved His_10_Smt3-tag was then separated from the 3CLpro protein using an Ni-NTA spin column (Qiagen) equilibrated with 0.6 ml NP Buffer and the 3CLpro proteins were collected in the flow-through. Buffer exchange was then performed into 50 mM Tris pH 6.5 using Amicon Ultra 10K Devices (Millipore), and protein concentration was determined by BCA assay.

The sequence of purified recombinant 3CLpro proteins was confirmed by mass spectrometry analysis of excised protein bands from Coomassie Blue stained protein gels.

### Preparation of HRV-3C protease

Purified (>90%) N-terminal His-tagged recombinant HRV-3C protease from Human Rhinovirus 14 (Sigma) was subjected to buffer exchange into 50 mM Tris pH 6.5 using Amicon Ultra 10K Devices (Millipore) and protein concentration determined by BCA assay.

### Cloning of 3CLpro and HRV-3C substrates

Codon-optimised complementary DNA oligonucleotides encoding protease substrate cleavage sites for 3CLpro (TSAVLQSGFRCL) (3CL-Sub-His F and R) and HRV-3C (LEVLFQGPCL) (HRV3C-Sub-His F and R) with a C-terminal His_6_ tag for expression in *E. coli* and flanked by 5′ BamHI and 3′ XhoI cohesive ends were synthesised (Sigma) (Table 1) and annealed in Annealing Buffer (10 mM Tris pH 8.0, 50 mM NaCl, 1 mM EDTA). The annealed DNA oligonucleotides were then ligated with BamHI/XhoI-digested pET28-His_10_Smt3. The resulting pET28-3CLpro and HRV-3C-Sub-His plasmids encode 17 kDa 3CLpro and HRV-3C substrates with an N-terminal His_10_Smt3 tag and a C-terminal His_6_ tag.

### Expression and purification of 3CLpro and HRV-3C substrates

BL21-CodonPlus (DE3)-RIPL *E. coli* competent cells (Agilent) were transformed with pET28-3CLpro and HRV-3C-Sub-His DNA. Cultures were grown at 30°C in 30 ml LB medium containing 30 µg/ml Chloramphenicol until OD_600_ reached 0.6 and then induced with 0.8 mM IPTG at 30°C for 3 h. The cells were harvested at 4000 rpm for 5 min and the pelleted cells were frozen at −20°C.

Frozen cell pellets were thawed on ice and resuspended in NP Buffer with 0.1% Triton X-100, 200 µg/ml Lysozyme and 25 U/ml Benzonase and incubated on ice for 30 min. Imidazole was then added to a final concentration of 10 mM and the cell lysates were separated at 17 000***g*** for 30 min. The cell lysates were applied to Ni-NTA spin columns (Qiagen) equilibrated with 0.6 ml NP, 10 mM Imidazole was then added. After two washes with 0.6 ml NP Buffer with 20 mM Imidazole, the His_10_Smt3-tagged 3CLpro- and HRV-3C-Sub-His proteins were eluted with 0.4 ml NP Buffer with 500 mM Imidazole. Buffer exchange was then performed into 50 mM Tris pH 6.5 using Amicon Ultra 10K Devices (Millipore) and protein concentration determined by BCA assay.

### Preparation of citrate-stabilised AuNP solution

Citrate-stabilised AuNP solution was prepared as previously described [19]. Briefly, 30.4 µl 1.445 M Gold(III) chloride (HAuCl_4_) solution (99.99% trace metals basis) (Sigma) was diluted in 99 ml distilled water and warmed to boiling point. Trisodium citrate dihydrate (Sigma) was then added to a final concentration of 0.59 mg/ml dropwise with vigorous stirring and incubated at boiling point for 15 min. The resulting red solution was cooled to room temperature to obtain the citrate-stabilised AuNP solution and stored at 4°C protected from light in a darkened bottle or wrapped in foil, although the solution is also stable at room temperature.

### Preparation of peptides

Native 3CLpro substrate peptide (H_2_N-EEEETSAVLQSGFRC-COOH) was synthesised by ThermoFisher Scientific and Optimised 3CLpro substrate peptide (H_2_N-EEEETHVVLQASFRC-COOH) was synthesised by Peptide Synthetics at >98% purity. The lyophilised peptides were dissolved in 37.6 mM Tris pH 8.0 at 2 mM peptide concentration.

### Protease assays

For the AuNP protease assays, variable concentrations of purified recombinant 3CLpro and HRV-3C proteases were incubated with 0.1 mM 3CLpro substrate peptide solution in the presence of 20 mM Tris pH 6.5, 50 µM EDTA, 10 µg/ml BSA, 100 mM NaCl and 0.05% NP-40 for 90 min at 37°C unless otherwise stated. 10 µl digested peptide solution was then added to 190 µl citrate-stabilised AuNP solution (unless otherwise stated) under normal laboratory conditions. After mixing, absorbance at 625 nm and 525 nm was measured on a microplate reader (Tecan Infinite 200 pro) and the A_625_/A_525_ ratio was calculated. Images were obtained on a PC scanner.

For 3CLpro and HRV substrate protein protease assays, 1 µg purified recombinant 3CLpro- and HRV-3C-Sub-His proteins were incubated with 400 nM purified recombinant SARS-CoV-2 or OC43 3CLpro or HRV-3C protease in the presence of 20 mM Tris pH 6.5, 50 µM EDTA, 10 µg/ml BSA, 100 mM NaCl and 0.05% NP-40 for 90 min at 37°C. Digested protease reactions were then analysed by SDS–PAGE Coomassie Blue Stain and Western Blot analysis.

### Size exclusion centrifugal filtration

Protease reaction samples were applied to Amicon Ultra 10K Devices (Millipore) and centrifuged at 14 000***g*** for 30 min at 4°C and flow-through was retained.

### SDS–PAGE coomassie blue stain and western blot analysis

Samples were denatured in 1× SDS–PAGE Loading Buffer (50 mM Tris 6.8, 2% SDS, 0.1% Bromophenol Blue, 10% glycerol, 25 mM DTT) at 95°C for 5 min. Denatured samples were separated on NuPAGE 4-12% Bis-Tris Mini Protein Gels (ThermoFisher Scientific).

For Coomassie Blue Stain, gels were stained with ProtoBlue Safe working solution (National Diagnostics) according to the manufacturer's instructions and imaged on Li-cor Odyssey Fluorescent Imaging System.

For Western Blot, wet transfer was performed onto Immobilon-FL PVDF membrane (Merck) and confirmed by reversible Ponceau S stain. Immunoblotting was performed using primary and secondary antibodies listed in Table 2 and imaged on Li-cor Odyssey Fluorescent Imaging System.

**Table 2:** A list of antibodies used in this study for Western blot analysis

Antibody	Species	Dilution	Source	Catalogue number
Anti-SARS-CoV-2 Nsp5 (3CLpro)	Sheep	1 : 500	MRC PPU reagents	Sheep Number: DA118, second bleed
Anti-SARS-CoV-2 Spike	Mouse	1 : 5000	GeneTex	632604
Anti-His-Tag	Mouse	1 : 1000	Cell signalling technologies	2366
Anti-His-Tag	Rabbit	1 : 1000	Cell signalling technologies	2365
Anti-β-Tubulin (TUBB)	Rabbit	1 : 1000	Cell signalling technologies	2146
Anti-Sheep IgG (H + L) Cross-Adsorbed Secondary Antibody, DyLight 800	Rabbit	1 : 15 000	Invitrogen	SA5-10060
Anti-rabbit IgG (H + L) (DyLight™ 800 4X PEG Conjugate)	Goat	1 : 15 000	Cell signalling technologies	5151
Anti-rabbit IgG (H + L) (DyLight™ 680 Conjugate)	Goat	1 : 15 000	Cell signalling technologies	5366
Anti-mouse IgG (H + L) (DyLight™ 800 4X PEG Conjugate)	Goat	1 : 15 000	Cell signalling technologies	5257

### Cell culture and transfection

HEK293T cells (ATCC CRL-3216) were cultured in DMEM supplemented with 10% FCS at 37°C in 5% CO_2_. Cells were seeded 24 h in advance of transfection at a density of 0.25 × 10^6^ cells per well of a six-well plate. pcDNA3.1 (control vector) or pcDNA3.1-Mpro (3CLpro expression vector) plasmids [32] were mixed at a ratio of 2.5 µg plasmid DNA: 2.5 µl Lipofectamine PLUS reagent: 7.5 µl Lipofectamine LTX reagent (Invitrogen) in 500 µl OptiMEM (Gibco). Cells were harvested 40 h post-transfection by scraping into lysis buffer (20 mM Tris pH 6.5, 50 µM EDTA, 100 mM NaCl and 0.1% NP-40) and incubated on ice for 15 min. Insoluble cellular debris was collected by centrifugation at 10 000***g*** at 4°C and supernatant was retained as cellular lysate. Cellular lysates were analysed immediately by protease assays and by SDS–PAGE and Western Blot.

### Production and titration of SARS-CoV-2 viral stocks

The virus used in this study was the lineage B viral isolate SARS-CoV-2/human/Liverpool/REMRQ0001/2020, a kind gift from Ian Goodfellow (University of Cambridge), isolated early in the COVID-19 pandemic by Lance Turtle (University of Liverpool) and David Matthews and Andrew Davidson (University of Bristol) from a patient from the Diamond Princess cruise [41–43].

Viral stocks were typically prepared by passaging once in VeroE6 cells. In brief, cells were infected at a low MOI with the original viral stock and incubated for 72 h (by which time cytopathic effect was evident). Virus-containing culture supernatants were then clarified by centrifugation at 600***g*** for 5 min and immediately frozen in aliquots at −80°C. For the determination of multiplicity of infection (MOI), viral stocks were titrated in VeroE6 cells by 50% tissue culture infectious dose (TCID50) assays using standard methods.

### 3CLpro expression during SARS-CoV-2 infection

To determine 3CLpro protein expression levels in the context of infection with authentic SARS-CoV-2, permissive HEK293T cells over-expressing the cellular SARS-CoV-2 receptor ACE2 (HEK293T-ACE2 cells [32]) were seeded in six-well plates at 80% confluence, then infected the following morning with SARS-CoV-2 at the indicated MOI. After 36 h, infected cells were washed once with PBS, then lysed in 100 µl/well lysis buffer (RIPA, Sigma–Aldrich Cat no R0278) supplemented with protease inhibitors (Halt Protease Inhibitor Cocktail, Thermo Fisher Cat no 7786) for 30 min at room temperature with occasional swirling. 0.1 U/µl Benzonase (Merck Millipore Cat no 70746) was added and incubated on ice for 30 min to digest nucleic acids. Insoluble cellular debris was collected by centrifugation at 10 000***g*** at 4°C and supernatants were retained as cellular lysates. Lysates were frozen immediately, prior to analysis by SDS–PAGE and Western Blot.

### Statistical analysis

Net A625/A525 ratio was calculated by subtracting background A625/A525 ratio in the absence of protease. Normalised Net A625/A525 ratio was calculated by dividing net A625/A525 ratio by the maximum A625/A525 ratio minus the background A625/A525 ratio in the absence of protease.

Statistical tests were performed using GraphPad Prism 9 Software.

## Data Availability

All data have been included in the paper.

## Abbreviations

AuNP: gold nanoparticle
COVID-19: Coronavirus Disease 2019
Cys: Cysteine
HRV-14: human Rhinovirus Type 14
IMAC: immobilised metal ion affinity chromatography
MOI: multiplicity of infection
Nsp5: Non-structural protein 5
RT-PCR: Reverse-Transcription Polymerase Chain Reaction
SARS-CoV-2: Severe Acute Respiratory Syndrome Coronavirus 2
WT: wild-type
