# Supplementary material for: Development of a colorimetric assay for the detection of SARS-CoV-2 3CLpro activity
Source: Biochem J. 2022 Apr 21;479(8):901–20. doi: 10.1042/BCJ20220105 (PMC9162461; doi:10.1042/BCJ20220105)
Supplement: Supplementary Material [file BCJ-479-901-s1.pdf]

Supplementary Figure Legends

Figure S1: Purification of recombinant SARS-CoV-2 3CLpro wildtype and H41A and H163A mutant proteins.

Expression of recombinant SARS-CoV-2 3CLpro wildtype (WT) and H41A and H163A mutant proteins was induced in BL21 (DE3) Codon-Plus RIL bacteria with 0.8 mM IPTG as a fusion protein with an N-terminal His-Smt3 domain (see 'Soluble Lysate' samples). The complete fusion protein was then purified (see 'Purified Eluate' sample) and digested with SUMO protease. The N-terminal His-Smt3 domain was then purified from the remaining C-terminal untagged recombinant SARS-CoV-2 3CLpro WT, H41A and H163A mutant proteins (see 'SUMO Flow-through' sample). Proteins were separated by SDS-PAGE and visualised by Coomassie Blue Total Protein stain (**A**) and Western blot (**B**).

Figure S2: Recombinant SARS-CoV-2 3CLpro activity is inhibited by Chymostatin protease inhibitor.

Treatment with Chymostatin significantly reduces recombinant SARS-CoV-2 3CLpro concentration-dependent increases in A625/A525 ratio (error bars represent means ± standard deviation; *n*=3 independent experiments; **** P<0.0001 by 2-way ANOVA Sidak's Multiple Comparisons Test). A representative visual image from independent replicate experiments is shown in a panel beside the graph.

Figure S3: Purification of recombinant OC43 3CLpro protein.

Expression of recombinant OC43 3CLpro protein was induced in BL21 (DE3) Codon-Plus RIL bacteria with 0.8 mM IPTG as a fusion protein with an N-terminal His-Smt3 domain (see 'Soluble Lysate' samples). The complete fusion protein was then purified (see 'Purified Eluate' sample) and digested with SUMO protease. The N-terminal His-Smt3 domain was then purified from the remaining C-terminal untagged recombinant OC43 3CLpro protein (see 'SUMO Flow-through' sample). Proteins were separated by SDS-PAGE and visualised by Coomassie Blue Total Protein stain (**A**) and Western blot (**B**).

Figure S4: Purification of recombinant 3CLpro and HRV-3C protease substrate protein.

(**A**) A schematic diagram showing the design of the recombinant 3CLpro and HRV-3C substrate proteins with the cleavage site denoted by ^ (A). Expression of recombinant 3CLpro and HRV-3C substrate protein was induced in BL21 (DE3) Codon-Plus RIL bacteria with 0.8 mM IPTG as a fusion protein with an N-terminal His-Smt3 domain (see 'Soluble Lysate' samples). The complete fusion protein was then purified (see 'Purification Eluate' sample) and concentrated with buffer exchange (see Concentrate Eluate). Proteins were separated by SDS-PAGE and visualised by Coomassie Blue Total Protein stain (**B**) and Western blot (**C**).

Figure S5: Recombinant SARS-CoV-2 and OC43 3CLpro and HRV-3C proteases are catalytically active.

(**A**) 1 µg purified recombinant 3CLpro and HRV-3C substrate protein was separated by SDS-PAGE and total protein visualised with Coomassie Blue stain; in *in vitro* protease assays, 1 µg recombinant 3CLpro substrate protein was only cleaved in the presence of 400 nM recombinant SARS-CoV-2 and OC43 3CLpro protease and conversely 1 µg recombinant HRV-3C substrate protein was only cleaved in the presence of 400 nM recombinant HRV-3C protease. *In vitro* protease reactions were separated by SDS-PAGE and visualised by Coomassie Blue Total Protein stain (**B**) and Western blot (**C**).

Figure S6: Increasing the concentration of 3CLpro substrate peptide increases the rate of protease reaction.

Increasing the concentration of native 3CLpro substrate peptide from 50 µM (yellow points) to 100 (grey points), 200 (orange points) and 400 µM (blue points) significantly increases the rate of increase in net A625/A525 ratio with 150 nM recombinant SARS-CoV-2 3CLpro (error bars represent means ± standard deviation; *n*=3 independent experiments; * P<0.05, ** P<0.01, *** P<0.001, **** P<0.0001 by 2-way ANOVA Tukey's Multiple Comparisons Test). A representative visual image from independent replicate experiments is shown in a panel below the graph.

Figure S7: Increasing the concentration of 3CLpro substrate peptide improves the sensitivity of the AuNP protease assay.

Increasing the concentration of native 3CLpro substrate peptide from 100 µM (grey bars) to 200 (orange bars) and 400 µM (blue bars) significantly increases the net A625/A525 ratio at 50 nM to 100 nM recombinant SARS-CoV-2 3CLpro after 90 minutes (error bars represent means ± standard deviation; *n*=3 independent experiments; * P<0.05, ** P<0.01, **** P<0.0001 by 2-way ANOVA Tukey's Multiple Comparisons Test). A representative visual image from independent replicate experiments is shown in a panel below the graph.

Figure S8: SARS-CoV-2 3CLpro activity is maximal at 37°C.

Increasing the protease reaction incubation temperature from 23°C (blue points) to 30°C (orange points) and 37°C (grey points) significantly increases net A625/A525 ratio at 100 nM recombinant SARS-CoV-2 3CLpro after 90 minutes but net A625/A525 ratio is significantly reduced at 44°C (yellow points) (error bars represent means ± standard deviation; *n*=3 independent experiments; * P<0.05, ** P<0.01, *** P<0.001, **** P<0.0001 by 2-way ANOVA Tukey's Multiple Comparisons Test). A representative visual image from independent replicate experiments is shown in a panel below the graph.

Figure S9: Expression of 3CLpro in SARS-CoV-2-infected cell lysates.

3CLpro protein is expressed at lower levels in SARS-CoV-2-infected HEK293T-ACE2 cells than in HEK293T cells transiently transfected with 2.5 µg pcDNA3.1-Mpro plasmid (3CLpro expression vector). Proteins were separated by SDS-PAGE and visualised by Western blot. Densitometric quantification is shown below the blot relative to TUBB loading control and normalised to the HEK293T pcDNA3.1-Mpro Transfection sample (%). MOI, multiplicity of infection.

Figure S1

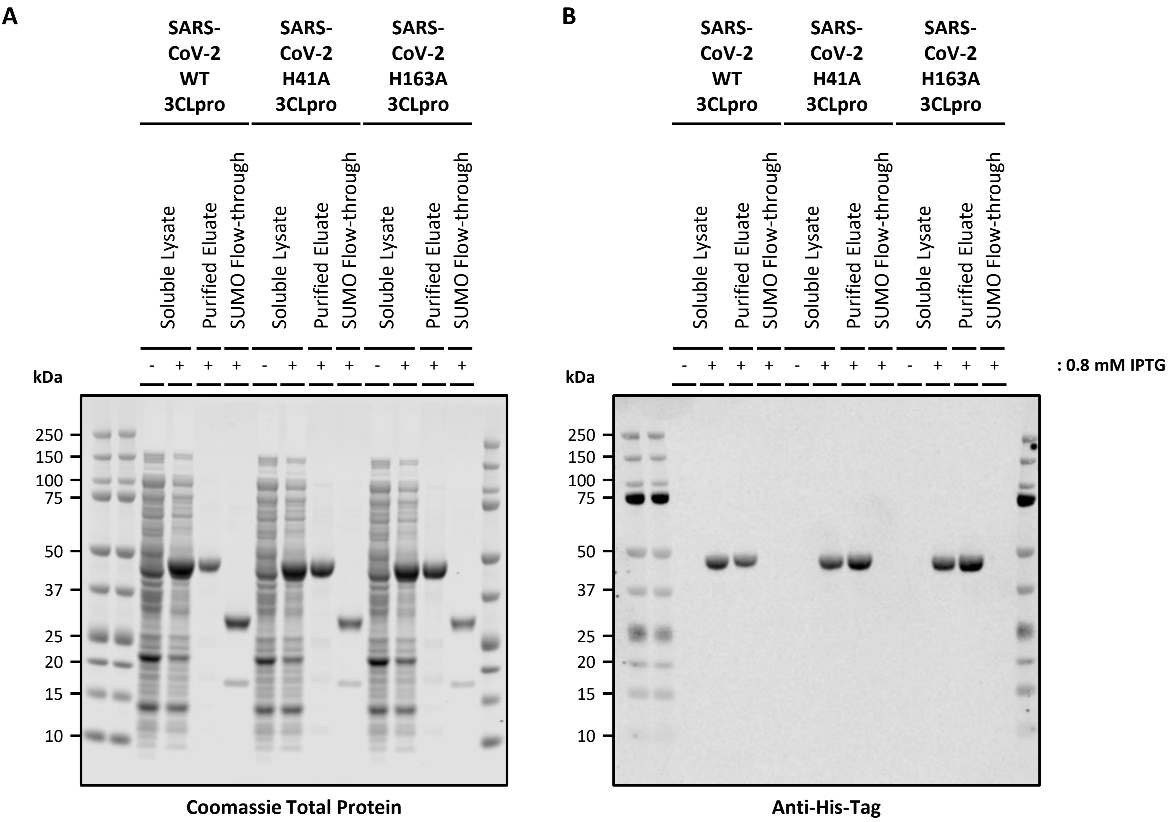
A
SARS-CoV-2 WT 3CLpro
SARS-CoV-2 H41A 3CLpro
SARS-CoV-2 H163A 3CLpro
Soluble Lysate
Purified Eluate
SUMO Flow-through
- + + +
kDa
250
150
100
75
50
37
25
20
15
10
Coomassie Total Protein
B
SARS-CoV-2 WT 3CLpro
SARS-CoV-2 H41A 3CLpro
SARS-CoV-2 H163A 3CLpro
Soluble Lysate
Purified Eluate
SUMO Flow-through
: 0.8 mM IPTG
Anti-His-Tag

Figure S2

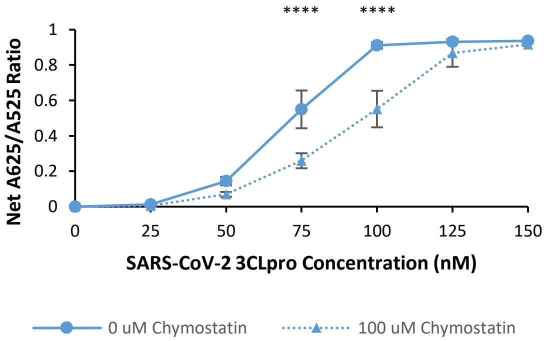

****
****
Net A625/A525 Ratio
1
0.8
0.6
0.4
0.2
0
0
25
50
75
100
125
150
SARS-CoV-2 3CLpro Concentration (nM)
0 uM Chymostatin
100 uM Chymostatin

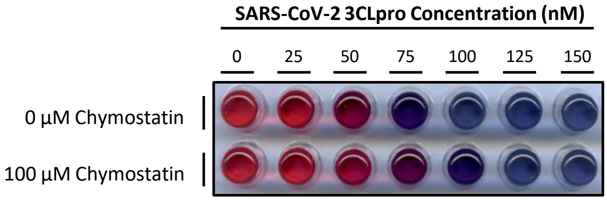

SARS-CoV-2 3CLpro Concentration (nM)
0
25
50
75
100
125
150
0 μM Chymostatin
100 μM Chymostatin

Figure S3

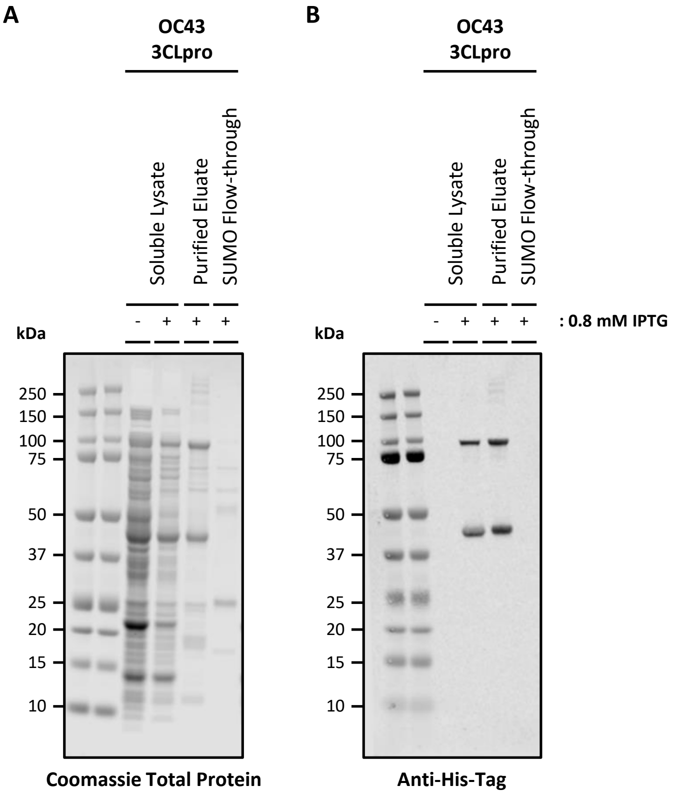
A
B
OC43
3CLpro
Soluble Lysate
Purified Eluate
SUMO Flow-through
- + + +
: 0.8 mM IPTG
kDa
250
150
100
75
50
37
25
20
15
10
Coomassie Total Protein
Anti-His-Tag

Figure S4

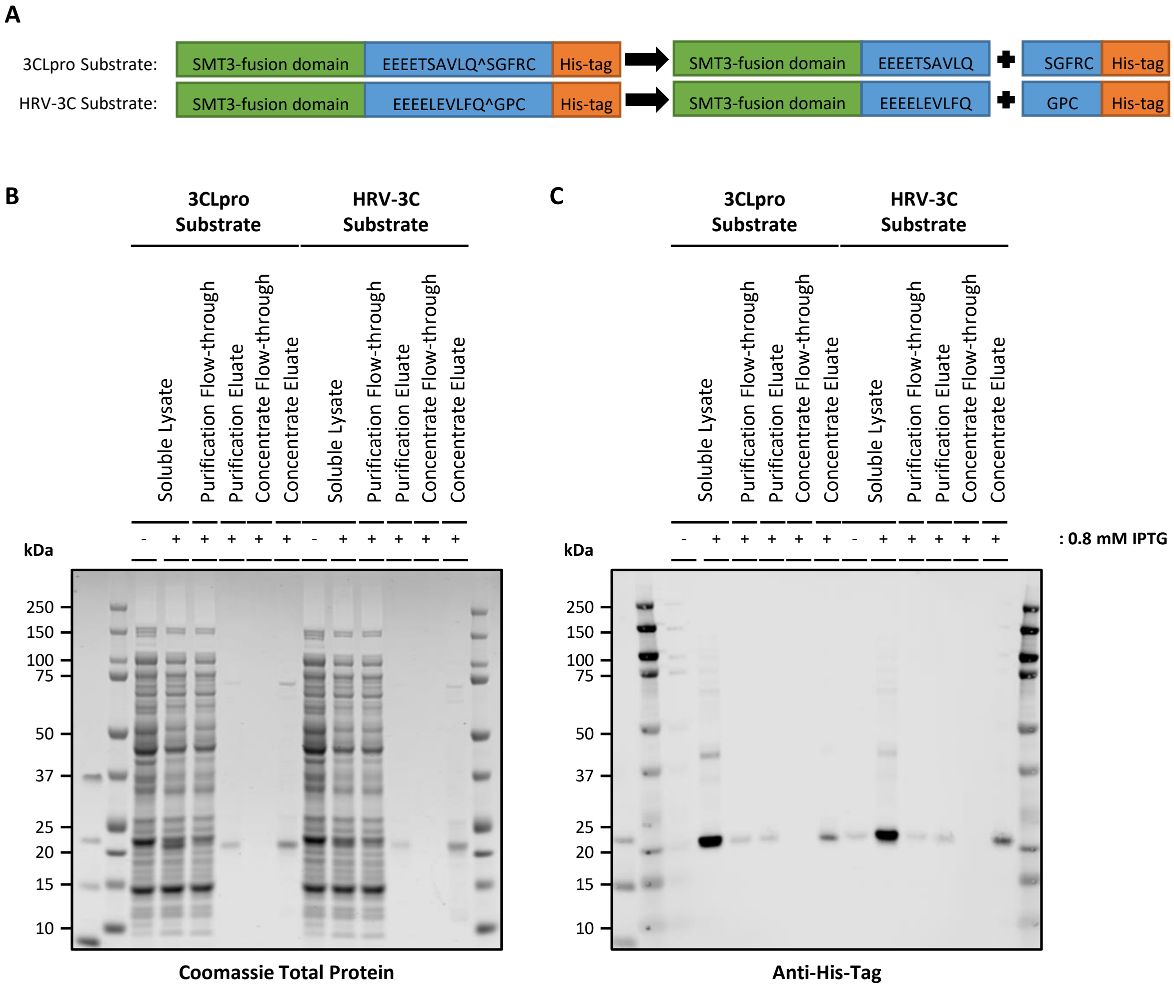

A
3CLpro Substrate:
SMT3-fusion domain
EEEETSAVLQ^SGFRC
His-tag
SMT3-fusion domain
EEEETSAVLQ
SGFRC
His-tag
HRV-3C Substrate:
SMT3-fusion domain
EEEELEVLFQ^GPC
His-tag
SMT3-fusion domain
EEEELEVLFQ
GPC
His-tag
B
3CLpro Substrate
HRV-3C Substrate
Soluble Lysate
Purification Flow-through
Purification Eluate
Concentrate Flow-through
Concentrate Eluate
Soluble Lysate
Purification Flow-through
Purification Eluate
Concentrate Flow-through
Concentrate Eluate
- + + + + + - + + + + +
kDa
250
150
100
75
50
37
25
20
15
10
Coomassie Total Protein
C
3CLpro Substrate
HRV-3C Substrate
Soluble Lysate
Purification Flow-through
Purification Eluate
Concentrate Flow-through
Concentrate Eluate
Soluble Lysate
Purification Flow-through
Purification Eluate
Concentrate Flow-through
Concentrate Eluate
- + + + + + - + + + + +
: 0.8 mM IPTG
kDa
250
150
100
75
50
37
25
20
15
10
Anti-His-Tag

Figure S5

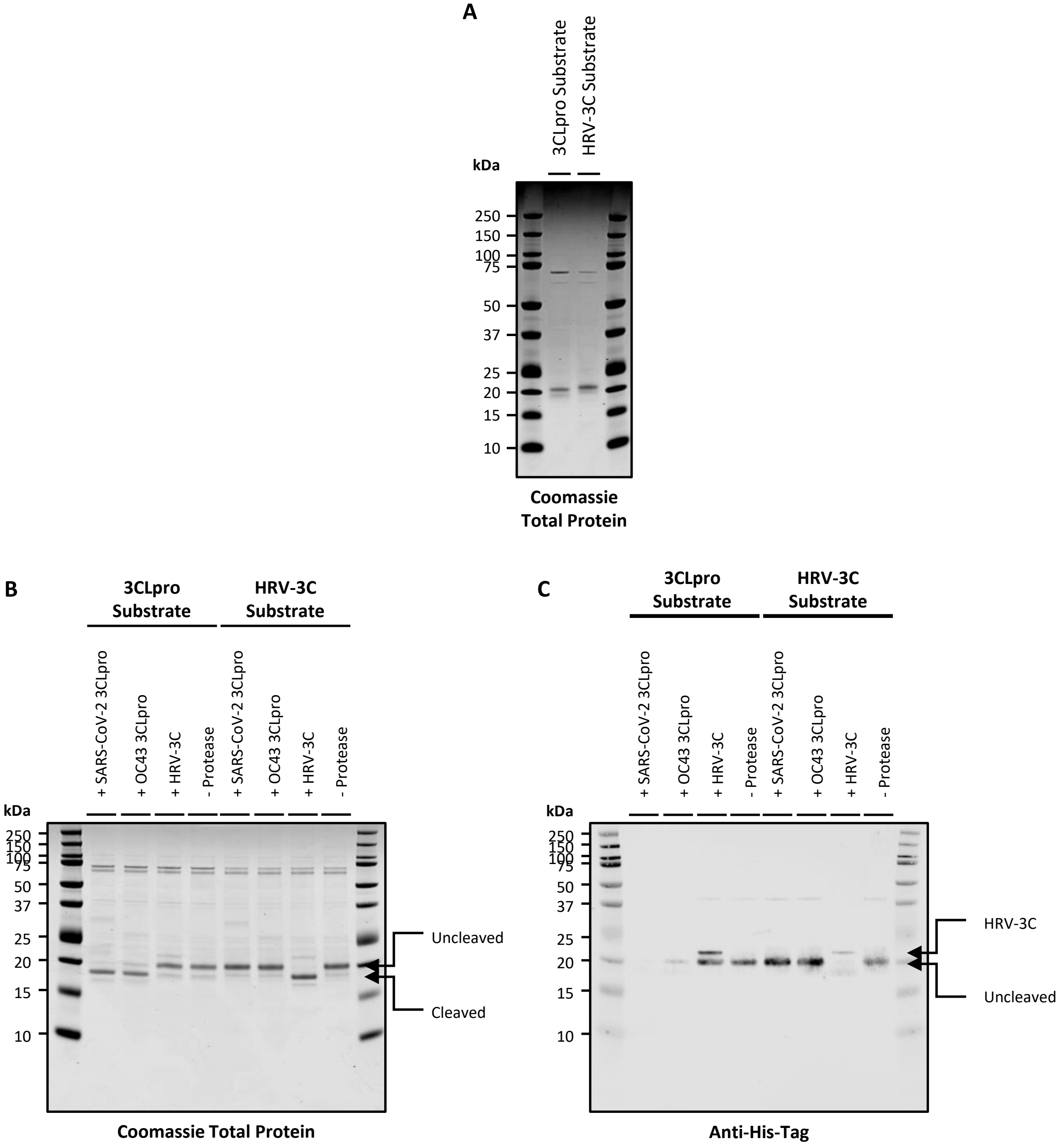
A
3CLpro Substrate
HRV-3C Substrate
kDa
250
150
100
75
50
37
25
20
15
10
Coomassie Total Protein
B
3CLpro Substrate
HRV-3C Substrate
+ SARS-CoV-2 3CLpro
+ OC43 3CLpro
+ HRV-3C
- Protease
Uncleaved
Cleaved
Coomassie Total Protein
C
HRV-3C
Uncleaved
Anti-His-Tag

Figure S6

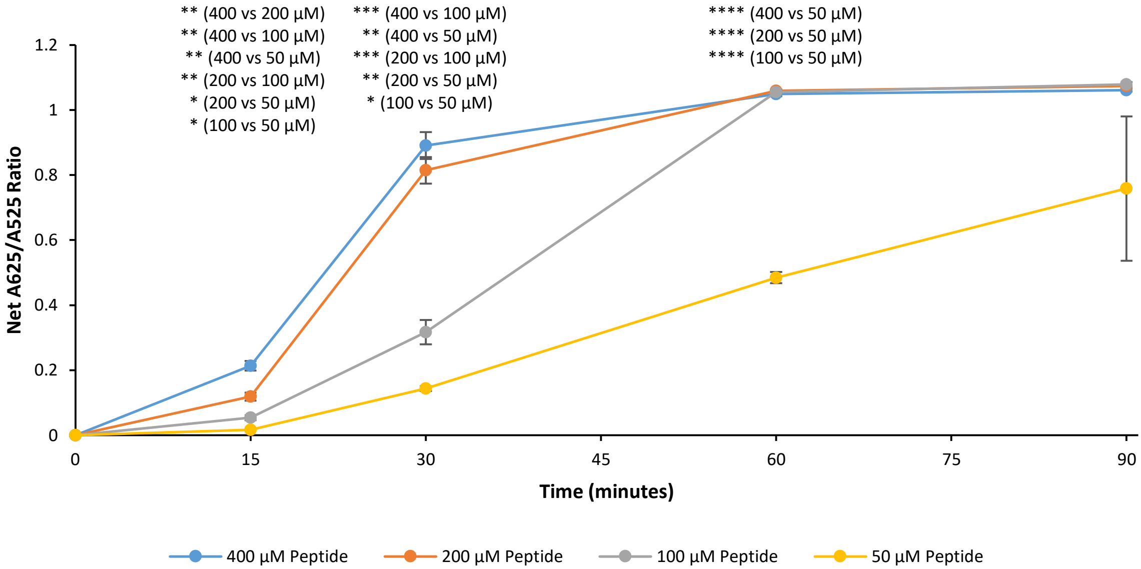

** (400 vs 200 µM)
** (400 vs 100 µM)
** (400 vs 50 µM)
** (200 vs 100 µM)
* (200 vs 50 µM)
* (100 vs 50 µM)
*** (400 vs 100 µM)
** (400 vs 50 µM)
*** (200 vs 100 µM)
** (200 vs 50 µM)
* (100 vs 50 µM)
**** (400 vs 50 µM)
**** (200 vs 50 µM)
**** (100 vs 50 µM)
Net A625/A525 Ratio
1.2
1
0.8
0.6
0.4
0.2
0
0
15
30
45
60
75
90
Time (minutes)
400 µM Peptide
200 µM Peptide
100 µM Peptide
50 µM Peptide

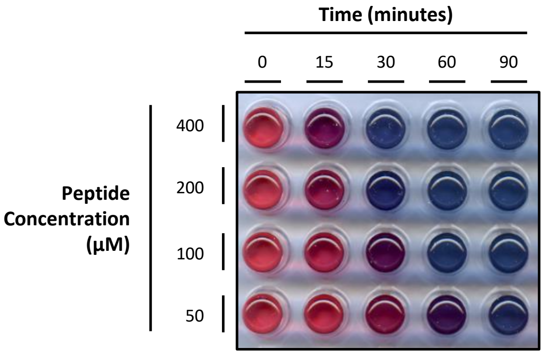

Time (minutes)
0
15
30
60
90
Peptide Concentration (µM)
400
200
100
50

Figure S7

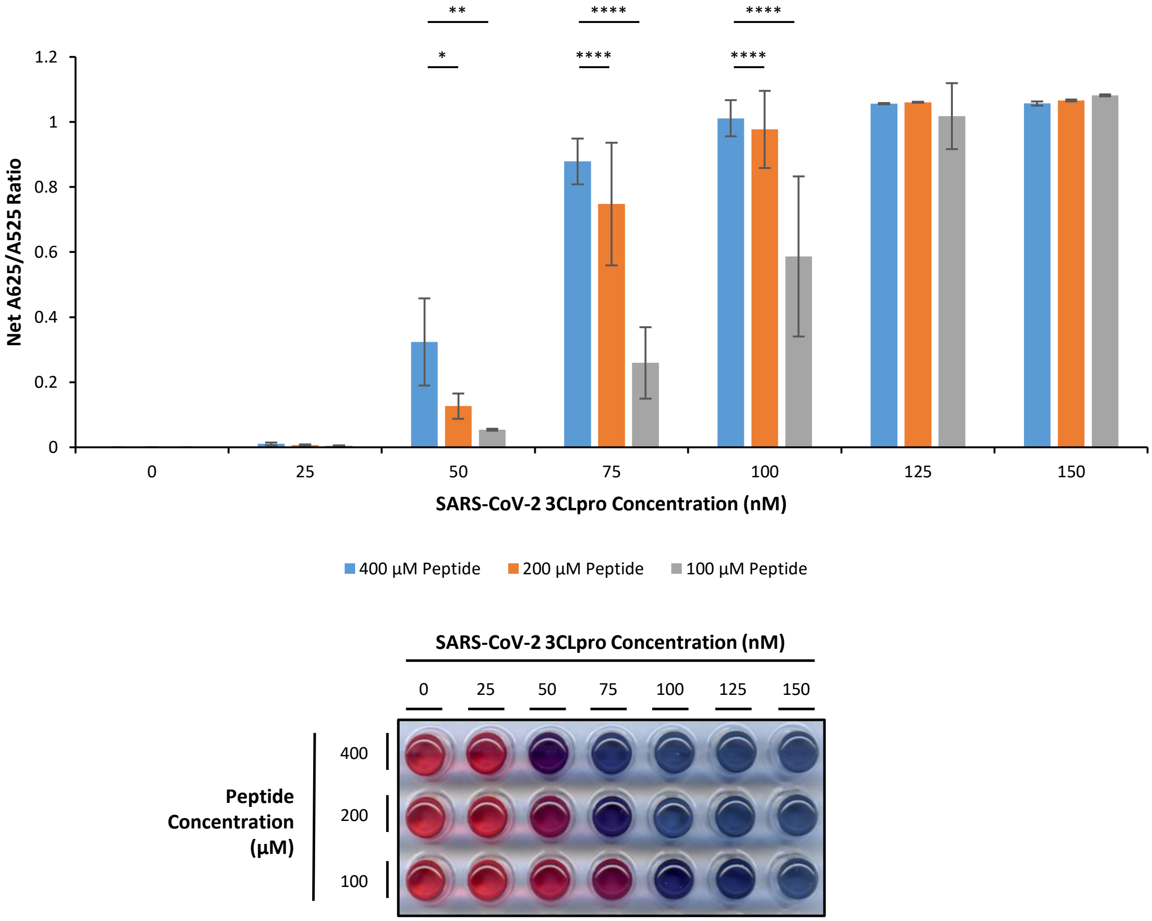
**
****
****
*
****
****
1.2
1
0.8
0.6
0.4
0.2
0
Net A625/A525 Ratio
0
25
50
75
100
125
150
SARS-CoV-2 3CLpro Concentration (nM)
400 µM Peptide
200 µM Peptide
100 µM Peptide
SARS-CoV-2 3CLpro Concentration (nM)
0
25
50
75
100
125
150
Peptide Concentration (µM)
400
200
100

Figure S8

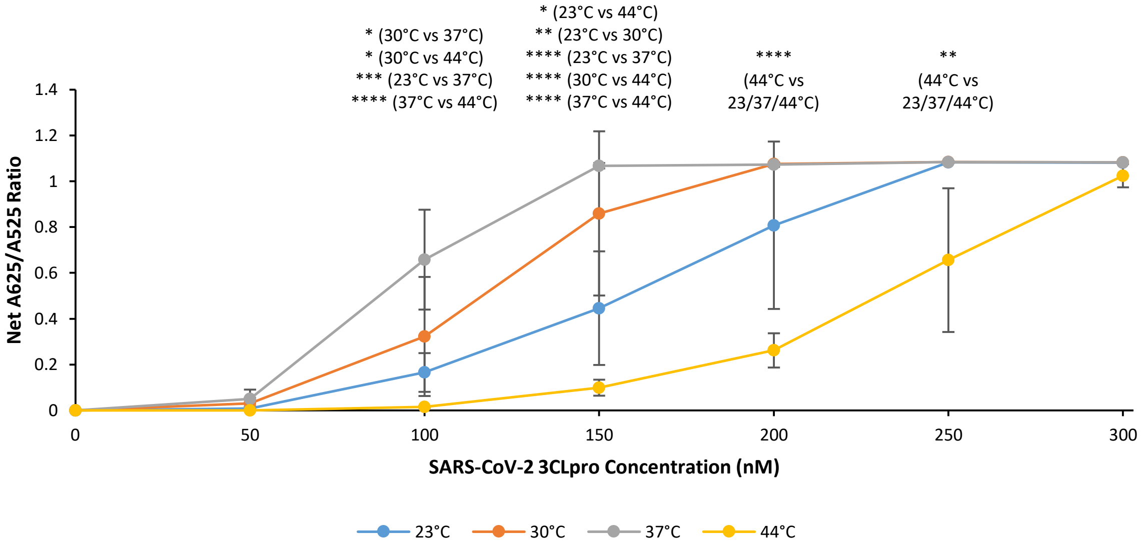

* (23°C vs 44°C)
** (23°C vs 30°C)
**** (23°C vs 37°C)
**** (30°C vs 44°C)
**** (37°C vs 44°C)
* (30°C vs 37°C)
* (30°C vs 44°C)
*** (23°C vs 37°C)
**** (37°C vs 44°C)
****
(44°C vs
23/37/44°C)
**
(44°C vs
23/37/44°C)
Net A625/A525 Ratio
0
0.2
0.4
0.6
0.8
1
1.2
1.4
0
50
100
150
200
250
300
SARS-CoV-2 3CLpro Concentration (nM)
23°C
30°C
37°C
44°C

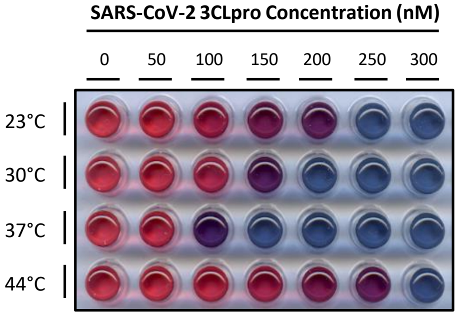

SARS-CoV-2 3CLpro Concentration (nM)
0
50
100
150
200
250
300
23°C
30°C
37°C
44°C

Figure S9

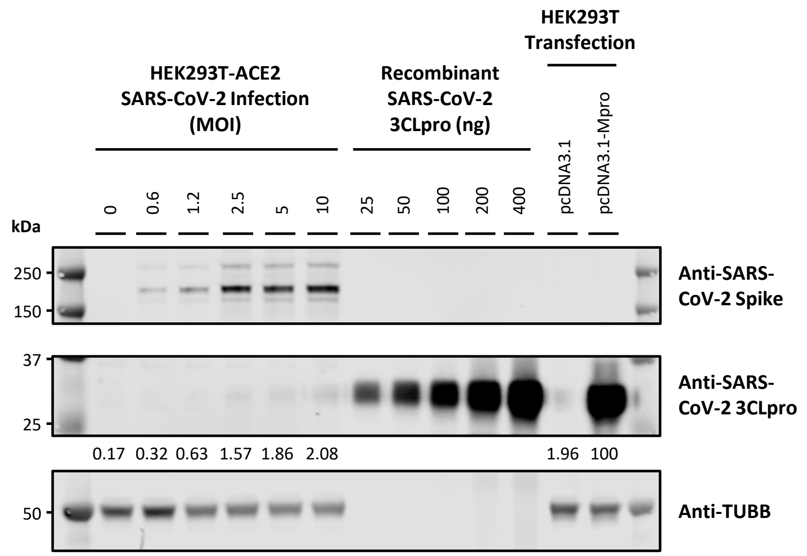
HEK293T Transfection
HEK293T-ACE2 SARS-CoV-2 Infection (MOI)
Recombinant SARS-CoV-2 3CLpro (ng)
pcDNA3.1
pcDNA3.1-Mpro
0
0.6
1.2
2.5
5
10
25
50
100
200
400
kDa
250
150
37
25
50
Anti-SARS-CoV-2 Spike
Anti-SARS-CoV-2 3CLpro
0.17 0.32 0.63 1.57 1.86 2.08
1.96 100
Anti-TUBB
